# *Clinacanthus nutans* extract lowers periodontal inflammation under high-glucose conditions via inhibiting NF-κB signaling pathway

**DOI:** 10.3389/fphar.2024.1410419

**Published:** 2024-08-13

**Authors:** Saruda Thongyim, Thomas A. Wright, Pachara Sattayawat, Thida Kaewkod, George S. Baillie, Yingmanee Tragoolpua, Siriphorn Jangsutthivorawat, Aussara Panya

**Affiliations:** ^1^ Doctor of Philosophy Program in Biology (International Program), Faculty of Science, Chiang Mai University, Chiang Mai, Thailand; ^2^ National Extracts and Innovative Products for Alternative Healthcare Research Group, Chiang Mai University, Chiang Mai, Thailand; ^3^ Office of Research Administration, Chiang Mai University, Chiang Mai, Thailand; ^4^ School of Cardiovascular and Metabolic Health, College of Veterinary Medical and Life Science, University of Glasgow, Glasgow, United Kingdom; ^5^ Department of Biology, Faculty of Science, Chiang Mai University, Chiang Mai, Thailand; ^6^ Cell Engineering for Cancer Therapy Research Group, Faculty of Science, Chiang Mai University, Chiang Mai, Thailand

**Keywords:** periodontal disease, inflammation, *Clinacanthus nutans*, herb extract, NF-κB signaling pathway

## Abstract

Periodontal disease is more prevalent in patients with diabetes, and it has a negative impact on their quality of life. Inhibiting the infection and inflammation processes that cause periodontal disease can reduce the severity of the disease and chances of serious complications. In this study, we aimed to demonstrate the effectiveness of *Clinacanthus nutans* extract in reducing the inflammation in gingival fibroblast cells induced by *Porphyromonas gingivalis* lipopolysaccharide (LPS). Stimulation with LPS under high-glucose conditions led to increased inflammation compared to low-glucose conditions. Treatment of *C. nutans* extract significantly reduced the expression of these pro-inflammatory cytokines and chemokines*.* At a concentration of 50 μg/mL, it reduced the relative expression of *IL6, IL8,* and *CXCL10* to 0.51 ± 0.09, 0.6 ± 0.19, and 0.09 ± 0.02, respectively, compared to the non-treatment control, accompanied by a decrease in secreted protein as measured by ELISA. Additionally, application of *C. nutans* extract markedly suppressed the NF-κB signaling pathway by reducing the phosphorylated form of IκBα, NF-κB p65, and nuclear translocation of NF-κB, along with a decrease in COX2, a key mediator in the inflammatory pathway. Furthermore, analysis of RNA sequencing data indicated that the extract clearly reversed the gene expression changes induced by LPS. This was particularly true for the signaling mediators and inflammatory genes in response to NF-κB, JAK/STAT, and TNF signaling pathways. Our finding highlights the potential of *C. nutans* extract to alleviate inflammation and suggests its potential as a treatment for periodontal disease in patients with diabetes.

## 1 Introduction

Periodontal disease is a chronic inflammatory disease caused by bacterial infections, including *Porphyromonas gingivalis*, *Aggregatibacter actinomycetecomitans*, *Prevotella intermedia*, and *Campylobacter rectus,* etc. (Arigbede et al., 2012; Shiga et al., 2020; Sedghi et al., 2021; Karched et al., 2022). The severity of this disease varies and can affect daily life with more serious cases resulting in tissue destruction. Periodontal disease can be classified based on the region of inflammation and its severity into two categories: gingivitis and periodontitis. Gingivitis is characterized by inflammation restricted to the gingiva and is reversible, whereas periodontitis is more severe, involving extended inflammation that leads to the irreversible destruction of the tissue and structure supporting the teeth.

The progression of periodontitis tends to be slow with approximately 40%–60% of reported cases being moderate, while severe cases affect 10%–15% of the adult population studied (Preshaw et al., 2012). The incidence and severity of periodontitis are correlated with factors such as smoking and certain medical conditions, including nutritional deficiency, osteoporosis, and the use of specific drugs which affect gingival overgrowth, i.e., calcium channel blockers, phenytoin, and ciclosporin (Preshaw et al., 2012). Notably, diabetes significantly increases the risk of developing periodontitis. Diabetic individuals face an approximately up to 3-fold higher risk of periodontitis compared to non-diabetic individuals (Mealey and Ocampo, 2007; Preshaw and Bissett, 2019) and this association can occur either in the type 1 or type 2 diabetes (Păunică et al., 2023). Research has shown a bidirectional interrelationships between diabetes and periodontitis (Păunică et al., 2023), wherein metabolic issues in diabetes increase the risk of periodontitis, while severe periodontitis can worsen diabetes-related complications such as retinopathy, diabetic neuropathy, proteinuria in addition to cardiovascular complications (Nguyen et al., 2020). Chronic inflammation plays a pivotal role in facilitating crosstalk between these conditions at both local and systemic levels. Massive cytokine production during chronic inflammation contributes to the interaction of periodontitis and diabetes, with common factors including IL-1β, IL-6, prostaglandin E2 (PGE2), TNF-α production (Preshaw et al., 2012). Levels of PGE2 and IL-1β in gingival crevicular fluid from diabetes patients with matched periodontitis severity were significantly higher than those in non-diabetic individuals (Polak and Shapira, 2018). Furthermore, the chemokine IL-8 and MIP-1β concentration in type 2 diabetes with chronic periodontitis were higher than those with chronic periodontitis group (Mohamed et al., 2015). This underscores the possibility that inflammation in diabetes exacerbates gingiva inflammation and contributes to disease progression. Consequently, targeting inflammation is a viable approach not only for treating periodontitis in diabetes patients but also for theoretically lowering the risk of non-oral complications associated with periodontitis.

Natural products are generally considered safe due to various factors, including their historical use and natural origins. Particularly, natural products listed in herbal pharmacopoeias are often deemed safe, as these documents indicate their long-reported use. Natural compounds derived from nature have garnered much attraction and can be used as therapeutics in the pharmaceutical industry. These compounds, known for their diverse biological activity and high safety profiles, are derived from the traditional use in folk medicine. Herb extracts, along with bioactive compounds, are now emerging as valuable sources for drug discovery. As mentioned, that in the case of *C. nutans*, it has been listed in Thai Herbal Pharmacopoeias which suggests its long use in Thailand. Our group previously reported the potential of *C*. *nutans* extract in reducing lipopolysaccharide (LPS)-induced cell death and inflammation in bovine endothelial cells (Panya et al., 2020; Thongyim et al., 2023). *Clinacanthus nutans* is a plant included in Thailand’s National List of Essential Herbal Medicines (2016) (Chaiprateep and Thavornwat, 2018) due to its traditional use in treating various health conditions. Particularly in Thailand, it has been used to treat viral infection, skin rashes and skin injuries such as snake and insect bites (Alam et al., 2016). This plant has been listed in the Thai Herbal pharmacopoeia in anti-inflammatory and antiviral categories. Additionally, the bioactivity of the plant’s compounds has been documented in numerous studies, demonstrating anti-inflammatory, antiviral, antioxidant, and anti-diabetic activities (Alam et al., 2016; Zulkipli et al., 2017; Nisar and Khan, 2020). Both the crude extract and a bioactive compound known as glyceryl 1,3-distearate (C_39_H_76_O_5_) demonstrated anti-inflammation properties by significantly reducing the gene expression of *IL1β, IL6, CXCL3,* and *CXCL8 (IL8)* upon LPS stimulation (Thongyim et al., 2023). We hypothesized that the anti-inflammatory potential of *C. nutans* extract could have a broad-spectrum effect, as bacterial LPS-induced inflammation in the bovine mastitis model and periodontal disease possibly shares common pathways. In this study, we aimed to investigate the potential of *C. nutans* extract in inhibiting inflammation in human gingival fibroblasts (HGF) cells upon stimulation by LPS from *P. gingivalis* in high-glucose conditions. The data presented here highlights the potential of *C. nutans* extract for further development in the treatment of periodontal disease for diabetes patients.

## 2 Materials and methods

### 2.1 *Clinacanthus nutans* and extraction

The fresh leaves of *Clinacanthus nutans* (Burm. f.) Lindau (cultured plants) were collected from Mae Rim District, Chiang Mai province in compliance with relevant institutional, national, and international guidelines and legislation. The plant specimens were identified by a botanist, Dr. Wittaya Pongamornkul, and deposited at the Queen Sirikit Botanic Garden Herbarium (QSBG herbarium) under a voucher number; WP8603. To comply with the ConPhyMP checklist (Heinrich et al., 2022), multiple chemical profile characterization methods should be employed. In this study, the *C. nutans* extract from the same source has been previously characterized through TLC, HPLC-UV/DAD, LC-MS/MS (Panya et al., 2020) and GC-MS/MS (Thongyim et al., 2023). It should be noted that direct overlay of the chromatograms with the commercial standards of potential bioactive compounds was performed to confirm the presence of the compounds. Moreover, we have performed the characterization of the extract thoroughly and to make sure that the extracts yield reproducing profiles. These analyses revealed several bioactive compounds, including glyceryl 1,3-distearate, identified as a glycolipid, and kaempferol 3-O-feruloyl-sophoroside 7-O-glucoside, identified as a C-glycoside flavone. These compounds are listed as active constituents in *C. nutans* according to the Thai Herbal Pharmacopoeia. Previously, the *C. nutans* used in this work has also been investigated using sequence-related amplified polymorphism (SRAP) technique in comparison with other sources of *C. nutans* in Thailand. The DNA patterns from seven pairs of SRAP markers for this *C. nutans* were reported (Chiangchin et al., 2023). The leaves of *C. nutans* were washed and dried at 60°C in a hot air oven for 72 h. The sample was ground to powder before resuspending in 70% ethanol at a ratio of 1:20 (w/v) and shaking at 160 rpm/min 25°C for 72 h. The total solution was evaporated in a rotary evaporator to collect the 70% ethanolic crude extracts. The 70% ethanolic crude extracts were dried in a chemical fume hood and stored at 20°C until use. According to the Thai Herbal Pharmacopoeia, the traditional method for preparing *C. nutans* extract involves crushing the leaves and soaking them in 40% (v/v) ethanol or Thai rice whiskey.

### 2.2 Antibacterial activity

The antibacterial activity was judged using a minimum inhibitory concentration (MIC) and a minimum bactericidal concentration (MBC). The MIC was conducted by the broth dilution method. *Clinacanthus nutans* extracts and glyceryl 1,3 distearate were prepared in Brain-heart infusion (BHI) broth (BD DifcoTM, Franklin Lakes, NJ, United States) at the indicated concentrations (two-fold dilution) where antibiotic gentamicin (Sigma-Aldrich, St. Louis, MO, United States) was used as a positive control. The bacterial suspension including *P. gingivalis*, *P. intermedia*, and *Streptococcus mutans* was adjusted for the turbidity to be matched with a 0.5 McFarland standard. The 50 µL of standardized bacteria suspension was added to 50 µL of prepared *C. nutans* extracts, glyceryl 1,3 distearate, or gentamycin in a 96-well plate. The plate was incubated at 37°C, under anaerobic conditions for 24 h. The lowest concentration with no visible growth after macroscopic evaluation was considered as MIC. After then, the culture was then streaked on BHI Agar (BD DifcoTM, Franklin Lakes, NJ, United States) and incubated at 37°C, under anaerobic conditions for a further 24 h. The lowest concentration of the plate with no visible growth was considered as MBC.

### 2.3 Cell line

HGF-1 cell line (Human Gingival Fibroblasts cells; ATCC®CRL-2014 Manassas, VA, United States) was cultured in the low glucose Dulbecco’s Modified Eagle Medium (DMEM; glucose 1 g/L) (Gibco, Thermo Fisher Scientific, MA, United States) supplemented with 10% (v/v) FBS and 1% (v/v) 200 mM L-glutamine (Gibco, Thermo Fisher Scientific, MA, United States) at 37°C in a 5% CO_2_ incubator.

### 2.4 Cell viability

The effect of LPS from *P. gingivalis* (InvivoGen, San Diego, CA, United States), *C. nutans* extract, and glyceryl 1,3 distearate on cell viability of HGF-1 cells was determined using cell viability assay. In brief, HGF-1 cells were plated a day before the experiment in 96-well plates (5,000 cells/well). The samples were prepared in media with low glucose (1 g/L) and high glucose (4.5 g/L) as followed concentrations; 0.78–100 μg/mL (2-fold dilution) of LPS derived from *P. gingivalis* (LPS-PG) or 4–2,500 μg/mL (5-fold dilution) of *C. nutans* extracts, or 0.63–20 μg/mL (2-fold dilution) of glyceryl 1,3 distearate (Sigma-Aldrich, St. Louis, MO, United States). The 100 µL of the sample was added to the cells and incubated for 24 h. The cell viability was measured by using PrestoBLUE™ cell viability reagent (Thermo Fisher Scientific, MA, United States) following the manufacturer’s protocol. The percentage of cell viability relative to the non-treatment control (set as 100%) was calculated as the equation below.

% Cell viability=OD570‐OD595 treated cells/OD570‐OD595 non‐treated cells×100

### 2.5 Real-time PCR

To determine the effect of glucose on inflammation induction, the HGF-1 cells were treated with the LPS-PG in the presence of low-glucose (1 g/L) and high-glucose (4.5 g/L) media. The HGF-1 cells were plated at 50,000 cells/well in 12-well plates 1 day before the experiment. The cells were treated with LPS-PG at concentrations of 0.1, 1, and 10 μg/mL, which were prepared in low-glucose and high-glucose media for 24 h. The inflammation in these two conditions was compared by monitoring the alteration of pro-inflammatory and inflammatory cytokines including IL-6, IL-8, and TNF-a judged by real-time PCR. In brief, total RNA was isolated using TRIzol^®^ reagent (Invitrogen, Carlsbad, CA, United States). The total RNA (500 ng) was used as a template for cDNA synthesis using Tetro cDNA Synthesis Kit (Bioline USA Inc., United States). Real-time PCR was then conducted in QIAquant real-time PCR cyclers (Qiagen, Venlo, Netherlands) using Luna^®^ Universal qPCR Master Mix (New England Biolabs, MA, United States) and specific primers (Supplementary Table S1). Fold changes of gene expression were calculated with the 2^−ΔΔCT^ method where *GADPH* was used as the housekeeping gene for the normalization of gene expression.

To measure the effect of *C. nutans* extract and glyceryl 1,3 distearate on lowering the expression of inflammatory genes, the cells were treated with LPS-PG at a concentration of 10 μg/mL in high-glucose media in the presence or absence of *C. nutans* extracts (25, 50, and 100 μg/mL) or glyceryl 1,3 distearate (5, 10, and 20 μg/mL). After 24 h of treatment, the cells were harvested followed by real-time PCR following the protocol described above.

### 2.6 Enzyme-linked immunosorbent assay (ELISA)

The HGF-1 cells were plated at 50,000 cells/well in 12-well plates 1 day before the experiment and the cells were treated with LPS-PG (10 μg/mL) in the absence or presence of *C. nutans* extract (100 μg/mL) in a high-glucose condition. The cells were incubated at 37°C in a 5% CO_2_ humidified atmosphere for 24 h. The culture supernatant was collected and used for determining the cytokine IL6 and IL8 by using a Human ELISA kit (ImmunoTools, Friesoythe, Germany). In brief, the capture antibody was added to the plate one night before the experiment at room temperature. The blocking buffer (1X PBS containing 2% bovine serum albumin and 0.05% Tween-20) was added to the well for 1 h. The buffer was removed and then the collected culture supernatant and the dilute standard protein were added to the wells. The plate was incubated at room temperature for 2 h before being washed with a wash buffer (1X PBS containing 0.05% Tween-20) for 5 times. The biotinylated detector antibody was added before adding the HRP-conjugated streptavidin. After 5-time washing, the 3–3′,5,5′-tetramethylbenzidine (TMB) substrate (Thermo Fisher Scientific, MA, United States) was added. After stopping the reaction with 2 N sulfuric acid, the absorbance was measured at OD450. The absorbance values were used to calculate the protein concentration relative to the corresponding standard protein curve.

### 2.7 Immunocytochemistry (ICC)

The HGF-1 cells were plated at 25,000 cells/well in 24-well plates 1 day before the experiment. The cells were treated with 10 μg/mL of LPS-PG in the absence or presence of *C. nutans* extract (100 μg/mL) in a high glucose media condition for 24 h. The treated cells were harvested and fixed with 3.6% formaldehyde for 15 min before being permeabilized with 0.2% Triton X for 15 min. The plate was blocked with 2% bovine serum albumin for 1 h before monoclonal antibody specific to NF-κB p65 clone D14E12 (dilution of 1:500, Cell Signaling, MA, United States) was added. The plate was incubated for 2 h followed by a washing step and the addition of a secondary antibody conjugated with Alexa-Fluor 488 (dilution of 1:1,000, Thermo Fisher Scientific, MA, United States) and Hoechst 33342 for nuclear staining (dilution 1:1,000, Thermo Fisher Scientific, MA, United States). The nuclear translocation of NF-κB protein was monitored under a confocal microscope (Nikon, Amsterdam, Netherlands), and NISelements AR program was used for fluorescence detection and image analysis.

### 2.8 Immunoblotting analysis

The cells were plated at 25,000 cells/well in 24-well plates 1 day before the experiment and were treated with LPS of *P. gingivalis* (10 μg/mL) in the absence or presence of *C. nutans* extract (300 μg/mL) in a high glucose media condition. The cells were incubated at 37°C in a 5% CO_2_ humidified atmosphere for 30 min to evaluate IKKα/β, IκBα, and NF-κB p65 and their phosphorylated forms whereas COX2 was detected at 24 h after treatment. The SDS-PAGE was performed using 12% polyacrylamide and then transferred to a nitrocellulose membrane. Membranes were blocked with 5% skim milk in 0.1%TBST for 1 h at room temperature before adding the monoclonal antibodies specific to phospho-IKKα/β, phospho-IκBα, and phospho NF-κB p65, COX2, GAPDH (Cell Signaling Technology, Boston, MA, United States) (1:1,000 dilution) followed by a goat anti-rabbit or mouse secondary antibody (ABclonal, Woburn, MA, United States) (1:1,000 dilution). The protein bands were detected using SuperSignal™ West Pico PLUS Chemiluminescent Substrate (Thermo scientific, IL, United States) and visualized by ImageQuant LAS 500 chemiluminescence CCD camera (GE, Boston, MA, United States). The membranes were stripped with 0.2 M NaOH for 3–5 min and were washed with 0.1%TBST for 5 min three times and used to detect IKKα/β, IκBα, and NF-κB p65 protein as the protocol described above. The protein band intensity was analyzed by ImageJ analysis software and normalized by GADPH (ABclonal, Woburn, MA, United States).

### 2.9 RNA sequencing

Total RNA extraction was carried out from HGF-1 cells in three different groups: DMSO-treated, LPS-treated, and LPS-treated in the presence of *C. nutans* extract (LPS + *C. nutans*) (n = 3), using Trizol reagent (Invitrogen, Carlsbad, CA, United States). Subsequently, samples from each group were combined. RNA sequencing was performed using the custom service of Novogene Corporation Inc. (Sacramento, CA, United States). mRNA was isolated using poly-T oligo-attached magnetic beads and fragmented using a fragmentation buffer. Random hexamers were added for reverse transcription to generate the first strand. Subsequently, the addition of dNTPs and DNA polymerase I resulted in the formation of double-stranded cDNA. The construction of the cDNA library consisted of a series of steps: end repair, A-tailing, adapter ligation, size filtering, USER enzyme digestion, amplification, and purification. Concentrations of the cDNA library were determined using both real-time PCR and the Qubit HS Assay kit (Thermo Fisher Scientific, Waltham, MA, United States), following the manufacturer’s instructions. The size distribution of the library was assessed using an Agilent Bioanalyzer (Agilent Technologies). Sequencing took place using an S4 flow cell and an Illumina NovaSeq 6000 (Illumina Inc.). Raw reads were processed to remove adaptor, poly-N and low-quality sequences. The resultant clean reads were aligned to the reference genome (GRCh38) using HISAT2 (v2.0.5). Using featureCounts (v1.5.0-p3) and gene annotations, gene expression was quantified and normalized as FPKM (Fragments per kilobase of exon model per million mapped reads). Next, DESeq2 (1.10.1) was used to analyze differential gene expression across the conditions. Genes were identified as differentially expressed if |log2(FoldChange)| > 1 and p.adj < 0.05. Following this, downregulated differentially expressed genes between the conditions of LPS + *C. nutans* and LPS were subjected to Gene Ontology (GO) enrichment analysis using the clusterProfiler package (version 4.6.2) in R. Enriched GO terms with a false discovery rate (FDR) < 0.05 were considered significant. Pathway analysis on the downregulated differentially expressed genes between the LPS + *C. nutans* and LPS conditions was carried out using KEGG analysis on the DAVID platform (https://david.ncifcrf.gov).

### 2.10 Statistical analysis

The bar graphs representing the mean and standard error of the mean (SEM) were plotted from three independent measurements. The statistical analyses were performed using Student’s *t*-test (Xing et al., 2024) of GraphPad Prism Software version 9 (GraphPad Software, Inc., La Jolla, CA, United States), where significant differences were indicated as follows (* indicates *p* < 0.05, **p < 0.01, ***p < 0.001).

## 3 Result

### 3.1 The antibacterial effect of *C. nutans* extract and glyceryl 1,3 distearate against periodontitis-causing anaerobic bacteria

The antibacterial activity of *C. nutans* has been previously reported but none of these reports have addressed its activity against anaerobic bacteria species that cause periodontitis. Therefore, we conducted novel experiments to assess the effect of *C. nutans* and its identified bioactive compound glyceryl 1,3 distearate, on inhibiting the growth inhibition of oral anaerobic bacteria including *P. gingivalis*, *P. intermedia*, and *S. mutans*. We treated the bacteria cultures with varying concentrations of *C. nutans* extract (ranging from 15.62 to 250 mg/mL) and glyceryl 1,3 distearate (ranging from 31.25 to 500 μg/mL) and determined their inhibition effects using minimum inhibitory concentration (MIC) and minimum bactericidal concentration (MBC) assays. The results indicated that both *C. nutans* extract and glyceryl 1,3 distearate had a limited impact on bacterial propagation (Table 1). The MIC and MBC for *C. nutans* extract against *P. gingivalis*, *P. intermedia*, and *S. mutans* was 250 mg/mL. Glyceryl 1,3 distearate exhibited less inhibitory effect, with MIC and MBC values of up to 500 μg/mL against *P. intermedia* and *S. mutans,* and 250 μg/mL against *P. gingivalis*. Notably, the positive control, gentamicin treatment, resulted in MIC and MBC values ranging from 7.81 to 31.25 μg/mL. When comparing the potency to glyceryl 1,3 distearate to gentamicin, gentamicin showed approximately 15.99-fold higher MIC against *P. gingivalis,* a 64.02-fold higher MIC against *P. intermedia,* and a 16-fold higher MIC against *S. mutans*. On the other hand, *C. nutans* extract exhibited more than a 1,000-fold higher MIC, demonstrating limited activity in controlling the growth of periodontitis-causing bacteria.

**TABLE 1:** Antibacterial activity of *C. nutans* extract and glyceryl 1,3 distearate against inhibiting the growth of oral bacteria associated with human periodontitis by MIC and MBC method.

	MIC and MBC					
Bacteria	*P. gingivalis*		*P. intermedia*		*S. mutans*	
	MIC	MBC	MIC	MBC	MIC	MBC
*C. nutans* extract (mg/mL)	250	250	250	250	250	250
Glyceryl 1,3 distearate (µg/mL)	250	250	500	500	500	500
^a^Gentamycin (µg/mL)	15.63	15.63	7.81	7.81	31.25	31.25

^a^ Positive control.

### 3.2 LPS derived from *P. gingivalis* (LPS-PG) strongly activated the inflammation response in human gingival fibroblasts-1 (HGF-1) cells

**FIGURE 1:**
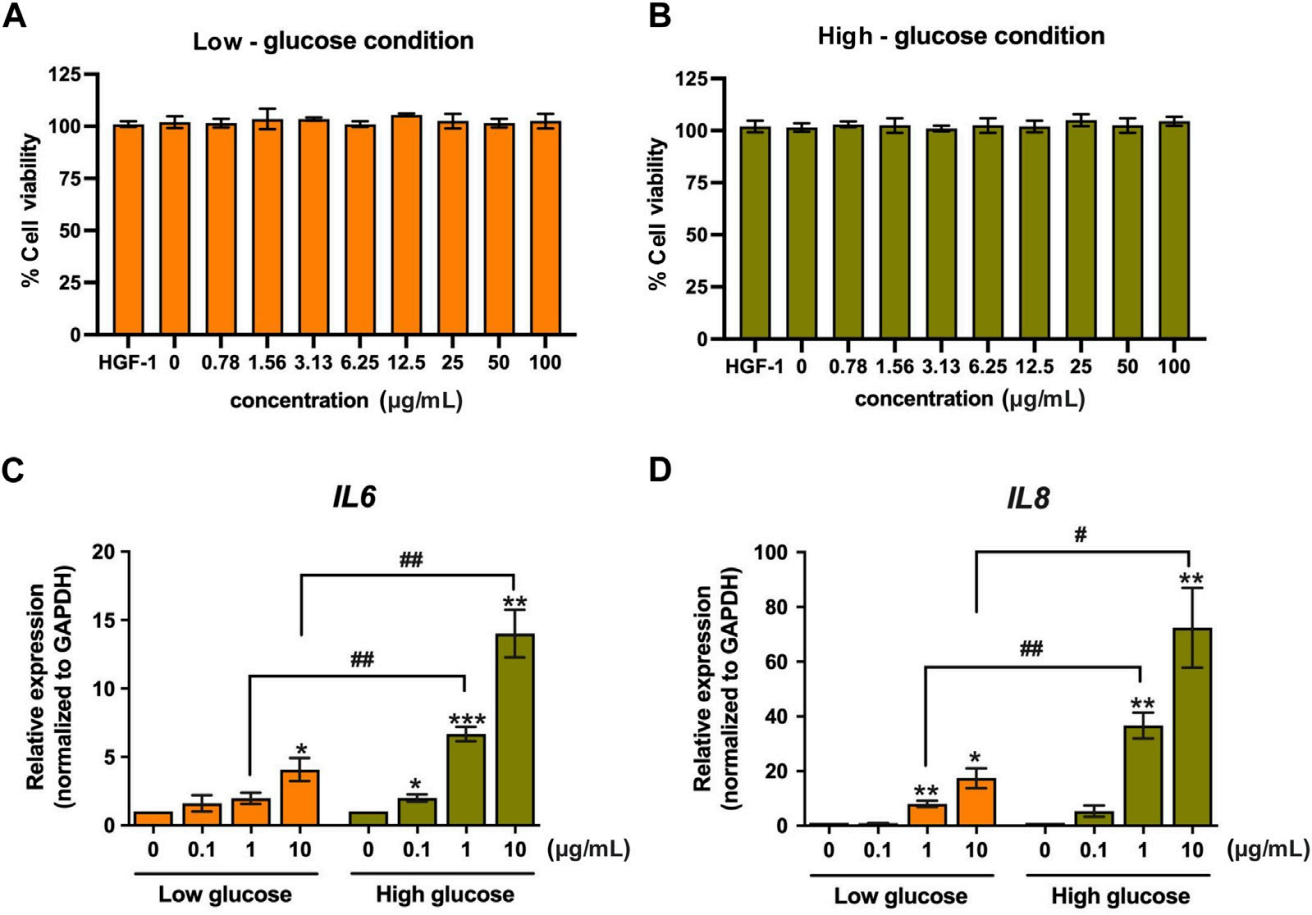
The effect of LPS on HGF-1 cells. Cytotoxicity of LPS-PG at concentrations of 0.78–100 μg/mL (2-fold dilution) in low-glucose **(A)** and high-glucose media **(B)** was monitored after 24-h treatment in HGF-1 cells by using cell viability assay. The changes in *IL6* **(C)** and *IL8* **(D)** mRNA expression after 24-h treatment of LPS (0.1, 1, and 10 μg/mL) were monitored using real-time PCR. The fold change of expression was normalized with *GAPDH* (housekeeping gene) and calculated as the relative expression to untreated cells *^/#^ indicates *p* < 0.05, ** or ^##^ *p* < 0.01, and *** or ^###^ *p* < 0.001. Values are expressed as means ± SEM from four replicates.

We investigated the effect of LPS-PG on the upregulation of key inflammatory gene expression (*IL6* and *IL8*) and its potential to induce cell death in HGF-1 cells. We compared two treatment conditions, high-glucose and low-glucose conditions, to assess the effect of LPS on individuals with and without diabetes. The result revealed that LPS treatment elicited an inflammation response but had no effect on cytotoxicity (Figure 1). Notably, following LPS treatment (0.78–100 μg/mL), the cell viability of HGF-1 cells remained consistently above 95% (Figure 1A). Expectedly, LPS treatment significantly induced an inflammatory response (Figure 1B). Treatment of the HGF-1 cells with LPS concentrations of 0.1, 1, 10 μg/mL resulted in the upregulation of mRNA expression of the inflammatory genes, *IL6* and *IL8.* Importantly*,* this upregulation was significantly pronounced under high-glucose conditions compared to low-glucose conditions. Under high-glucose conditions, a concentration of 10 μg/mL led to a remarkable 72.43-fold increase in mRNA expression of *IL8* and 14.02-fold increase in *IL6*, whereas under low-glucose conditions, these values were 17.42-fold and 4.08-fold, respectively (Figure 1B). These results suggest that LPS stimulation in high-glucose conditions exerts a strong induction of the inflammatory response in HGF-1 cells.

### 3.3 *C. nutans* extract and glyceryl 1,3 distearate lowered IL6 and IL8 mRNA expression after LPS stimulation

**FIGURE 2:**
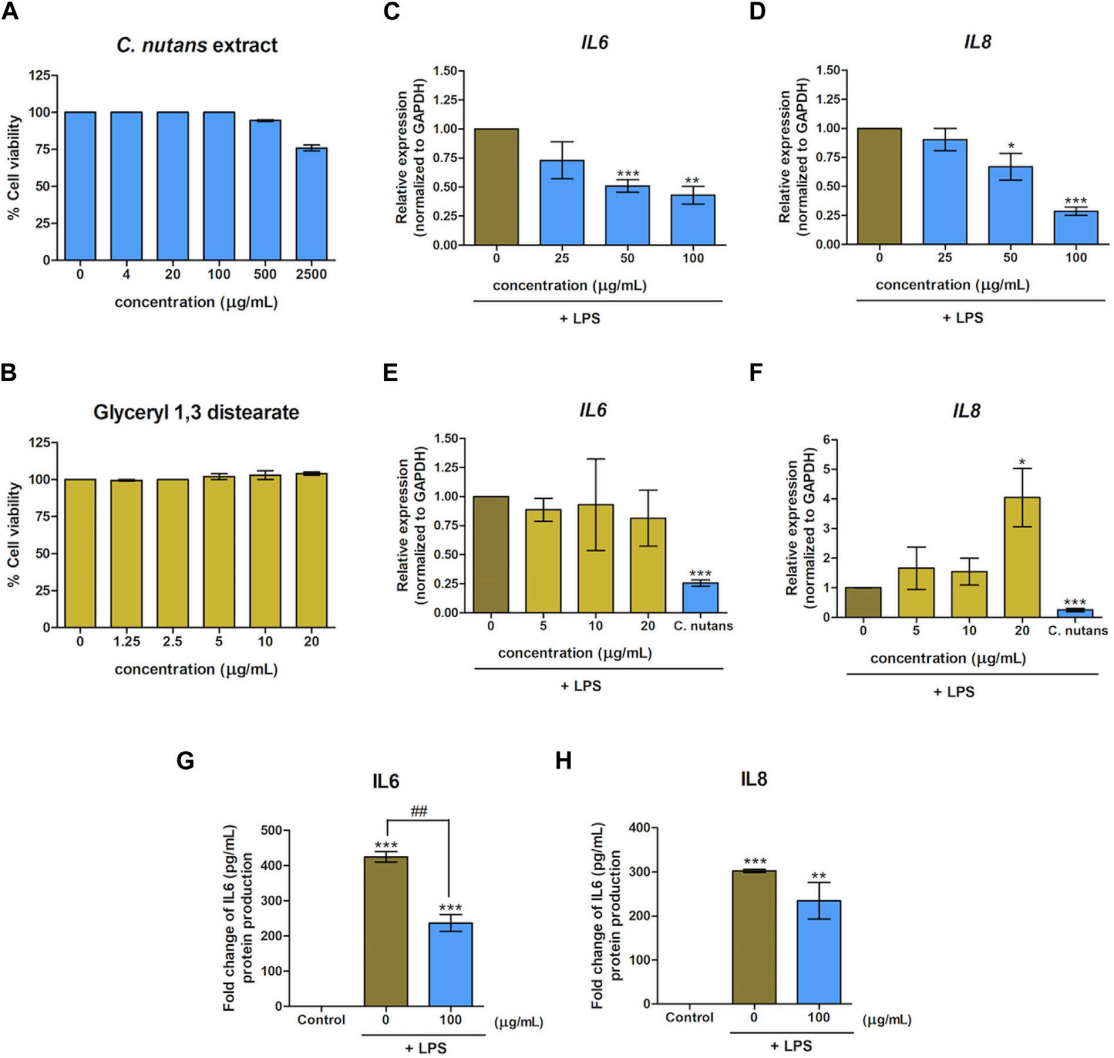
Effect of *C. nutans* extracts and glyceryl 1,3 distearate on lowering LPS-induced inflammation. Cytotoxicity of *C. nutans* extracts (4–2,500 μg/mL) **(A)** and glyceryl 1,3 distearate (0.63–20 μg/mL) **(B)** was determined by cell viability assay. The effects of *C. nutans* extracts **(C,D)** and glyceryl 1,3 distearate **(E,F)** on *IL6* and *IL8* mRNA expression after 24-h treatment of LPS (10 μg/mL) were monitored using real-time PCR. The fold change of expression was normalized with *GAPDH* (housekeeping gene) and calculated as the relative expression to untreated control (LPS alone). The effect of *C. nutans* extract (100 μg/mL) to reduce IL6 and IL8 proteins after LPS induction (10 μg/mL) **(G,H)**. * indicates *p* < 0.05, ** or ^##^ *p* < 0.01, and ***p < 0.001. Values are expressed as means ± SEM from three replicates.

In a previous study, we reported the anti-inflammatory activity of *C. nutans* extract and glyceryl 1,3 distearate in a bovine mastitis model (Thongyim et al., 2023). Building on this, we hypothesized that *C. nutans* extract and glyceryl 1,3 distearate would exhibit anti-inflammatory properties in HGF-1 cells following LPS stimulation. We conducted a cell viability assay to evaluate the toxicity of *C. nutans* extract and glyceryl 1,3 distearate on HGF-1 cells (Figures 2A,B). The results demonstrated that concentrations of up to 500 μg/mL of *C. nutans* extract and up to 20 μg/mL of glyceryl 1,3 distearate had no adverse effects on cell viability. Subsequently, we treated the cells with varying concentrations of *C. nutans* extract (25–100 μg/mL) and glyceryl 1,3 distearate (5–20 μg/mL) following LPS stimulation at a concentration of 10-µg/mL in high-glucose conditions. Real-time PCR analysis revealed that the *C. nutans* extract had a potent dose-dependent effect in reducing mRNA expression of *IL6* and *IL8* (Figures 2C,D). Treatment with a concentration of 100 μg/mL resulted in a significant reduction of relative expression, lowering *IL6* to 0.43-fold and *IL8* to 0.29-fold (Figures 2C,D). Conversely, glyceryl 1,3 distearate treatment showed no significant difference (Figures 2E,F). In summary, *C. nutans* extract, but not glyceryl 1,3 distearate, demonstrated potential in inhibiting inflammation induced by LPS stimulation.

Furthermore, we validated the inhibitory effect of *C. nutans* extract on the production of IL6 and IL8 at the protein levels. Upon LPS stimulation, the secreted levels of IL6 and IL8 increased significantly, reaching 424.21 pg/mL and 302.25 pg/mL, respectively. However, in the presence of *C. nutans* extract, these levels were markedly suppressed (Figures 2G,H). Specifically, the extract reduced IL6 to 236.74 pg/mL and IL8 level to 234.50 pg/mL, highlighting the effectiveness of *C. nutans* extract in attenuating LPS-induced inflammation under high-glucose conditions.

### 3.4 *C. nutans* extract inhibited LPS-induced inflammation through suppression of NF-κB signaling pathway

**FIGURE 3:**
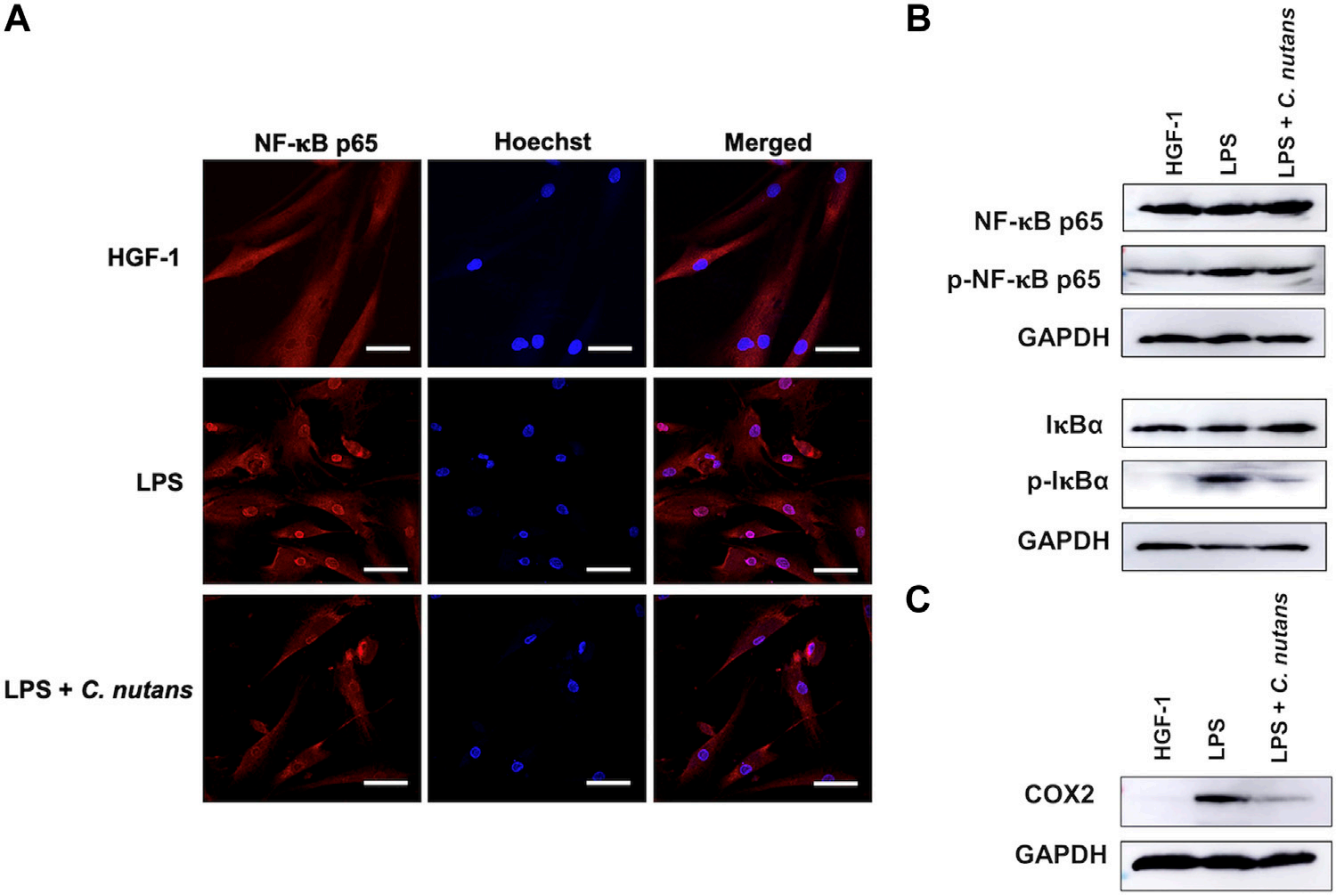
*C. nutans* extracts suppressed NF-κB signaling pathway. LPS (10 μg/mL) was treated to HGF-1 cells in the presence or absence of *C. nutans* extracts (300 μg/mL). NF-κB nuclear translocation was monitored by IFA techniques where the nuclear area was stained by Hoechst dye (NF-κB in red and nucleus in blue with 50-µm scale bar) **(A)**. Immunoblotting was performed to detect the changes of IκBα and NF-κB p65, phosphorylated IκBα, phosphorylated NF-κB p65 **(B)** and COX2 protein **(C)**. Photos are representatives from three replicates (Supplementary Figure S4).

The NF-κB signaling pathway is recognized as a key driver of inflammation, particularly in response of LPS stimulation. Consequently, we aimed to investigate whether the inhibitory effect of *C. nutans* extract operated through the NF-κB signaling pathway. HGF-1 cells were subjected to treatment with LPS (10 μg/mL) in the presence or absence of *C. nutans* extract (100-µg/mL). As expected, we observed a reduction in NF-κB nuclear translocation in cells treated with *C. nutans* extract compared to those subjected to LPS stimulation alone (Figure 3A). To further validate the effect of *C. nutans* extract on NF-κB signaling, we employed an immunoblotting technique. In agreement with the observed increase in NF-κB nuclear translocation, the levels of phosphorylated IκBα and NF-κB p65 were significantly elevated following LPS stimulation, but these increases were notably attenuated in the presence of *C. nutans* extract (Figure 3B).

Furthermore, we accessed changes in the protein levels of COX2, a pivotal regulator of an inflammatory process, after treatment with *C. nutans* extract in LPS-exposed cells (Figure 3C). LPS significantly increased the COX2 expression, validating the inflammatory response in the cells. However, the overexpression of COX2 was inhibited in the presence of *C. nutans* extract. Taken together, these results showed the effectiveness of *C. nutans* extract in attenuating inflammation by suppression of NF-κB signaling pathway.

### 3.5 Transcriptomic revealed *C. nutans* extracts inhibited inflammation and altered cytokine-mediated signaling pathways

**FIGURE 4:**
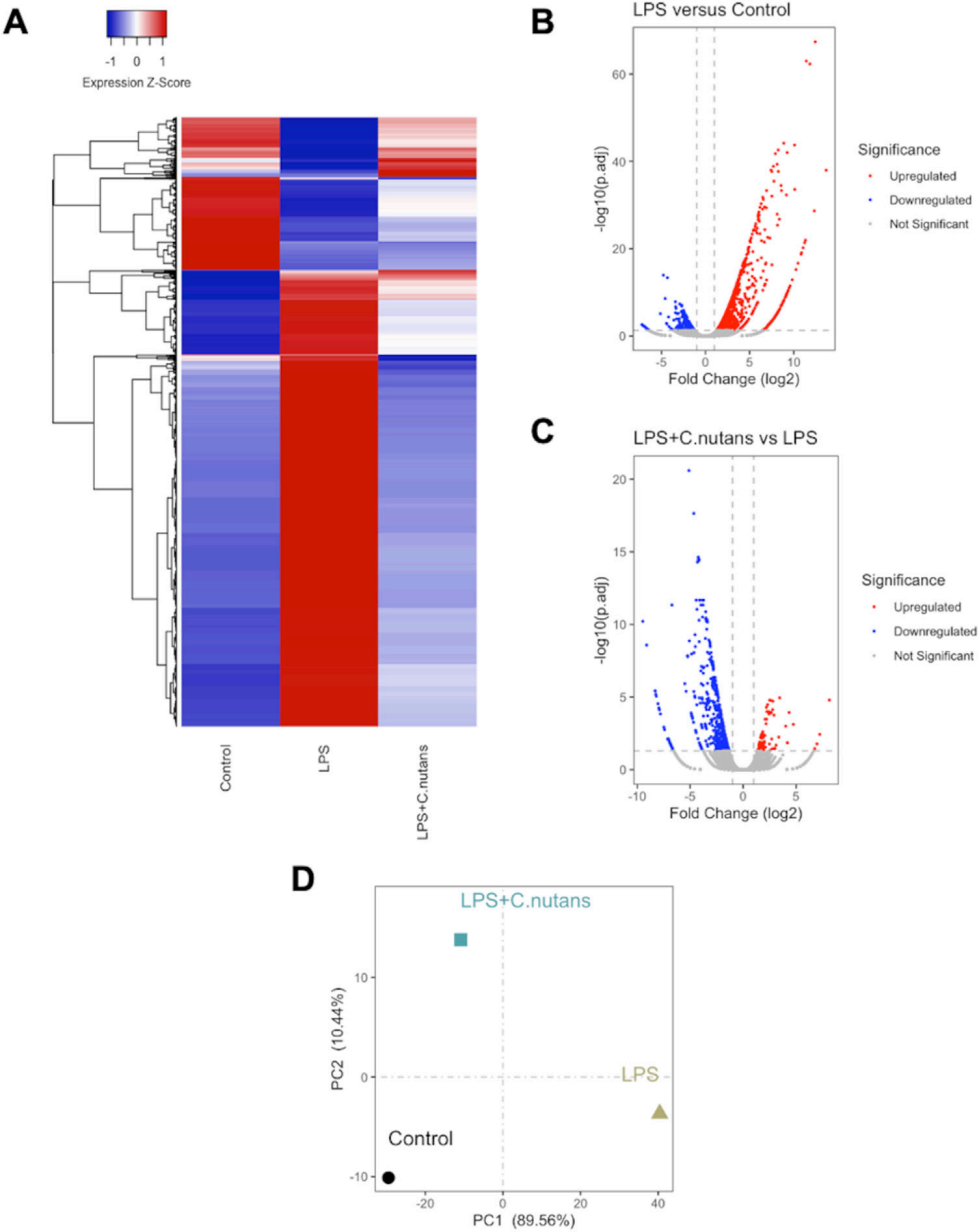
RNA-seq analysis of control cells, LPS-treated cells, and LPS + *C. nutans* treated cells. **(A)** Clustered heatmap showing scaled expression of DEGs across samples. **(B)** Volcano plot depicting DEGs between LPS-treated and control HGF-1 cells. **(C)** Volcano plot of DEGs between LPS + *C. nutans* treated and LPS-treated HGF-1 cells. **(D)** PCA analysis across samples. The variance in expression represented by principal components is indicated as a percentage.

To further assess the anti-inflammatory activity of *C. nutans* extract, we conducted RNA-seq analysis on HGF-1 cells subjected to different treatments: DMSO-treated control cells, LPS-treated cells (10 μg/mL for 24 h), and LPS-treated cells in the presence of *C. nutans* extract (100 μg/mL for 24 h). RNA sequencing was performed using the Illumina NovaSeq 6000 platform, generating over 40 million raw reads for each sample. Following trimming, we obtained over 40 million clean reads with a Q30 score exceeding 94%, indicating excellent data quality (Table 2). Subsequently, the reads were aligned to the reference genome, annotated, and quantified. The counts were then normalized as fragments per kilobase of exon model per million mapped reads (FPKM). Differential gene expression analysis was carried out using DESeq2, where genes with an adjusted *p*-value (p.adj) less than 0.05 and an absolute log2 fold change (|log2FC|) greater than 1 were considered statistically significant (Figures 4A–C). In comparison between LPS-treated cells and control cells, we identified 1,359 differentially expressed genes (DEGs) with 1,038 upregulated and 321 downregulated (Figure 4B). When comparing cells treated with LPS in the presence of *C. nutans* extract (LPS + *C. nutans*) to LPS-treated cells, we found 622 DEGs comprising 85 upregulated and 537 downregulated genes (Figure 4C). To evaluate the relationship in gene expression profiles among the three conditions, we performed the Principal Component Analysis (PCA) (Figure 4D). LPS treatment led to a substantial separation from the control cells along principal component 1, accounting for a total variance of 89.56%. However, when treating the LPS-exposed cells with *C. nutans* extract, this separation was reduced to cluster closer to the control cells. Along principal component 2, the cells treated LPS + *C. nutans* extract showed a slight separation from both the control and LPS-treated cells, which clustered together, accounting for 10.44% of the total variance. In summary, these finding suggest that LPS induced a significant perturbation in gene expression which is partially alleviated by *C. nutans* treatment.

**TABLE 2:** Read count and quality of raw and trimmed reads obtained from RNA-sequencing.

Sample	Raw reads	Trimmed reads	Q20 (%)	Q30 (%)
Control	46,246,964	45,787,292	97.91	94.05
LPS	4,6,741,926	4,6,291,766	97.98	94.16
LPS + *C. nutans*	41,517,532	40,915,622	97.97	94.11

**FIGURE 5:**
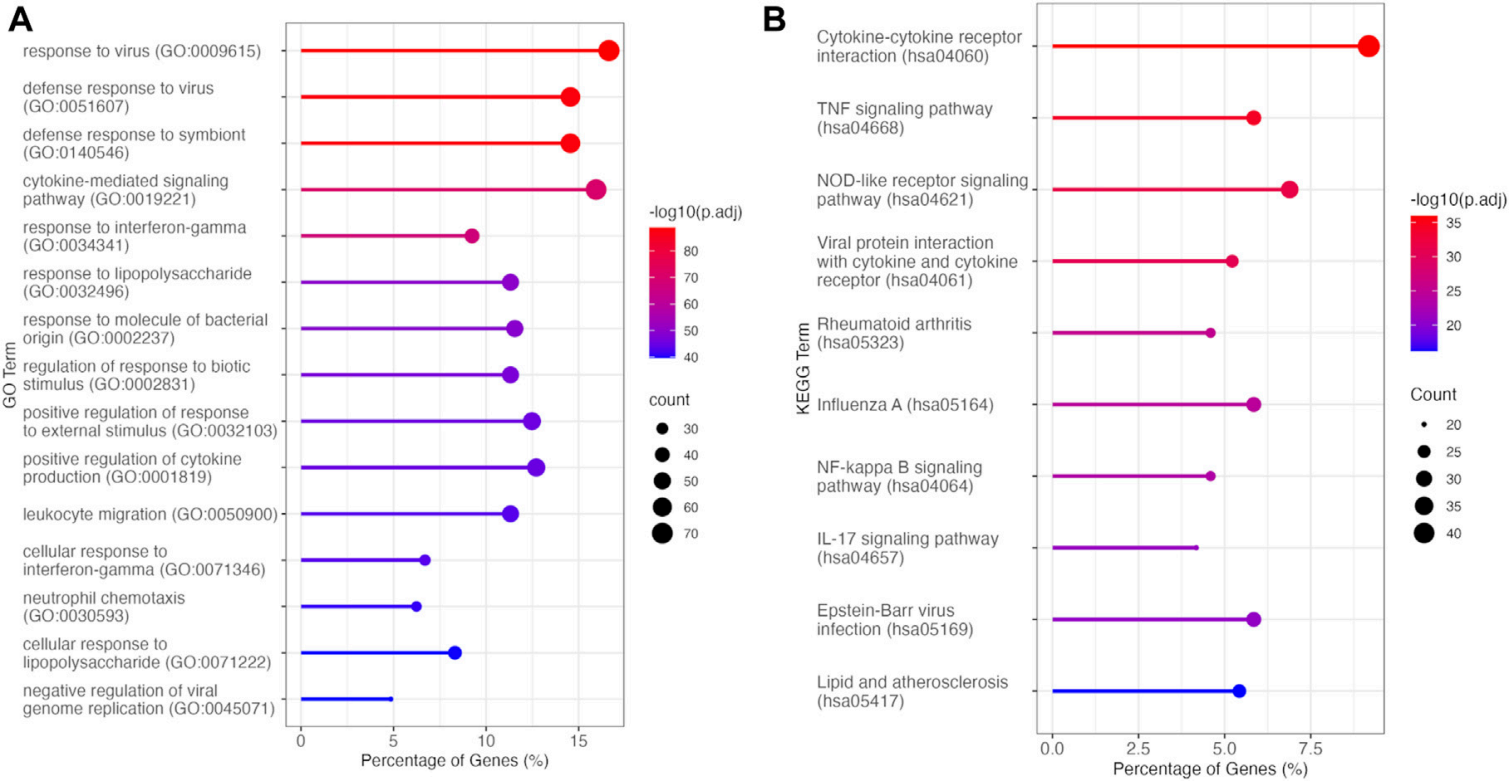
Overrepresentation analysis of DEGs downregulated between LPS + *C. nutans* and LPS treatments. **(A)** Top fifteen enriched processes identified by GO-BP analysis. **(B)** Top ten enriched pathways identified by KEGG analysis.

**FIGURE 6:**
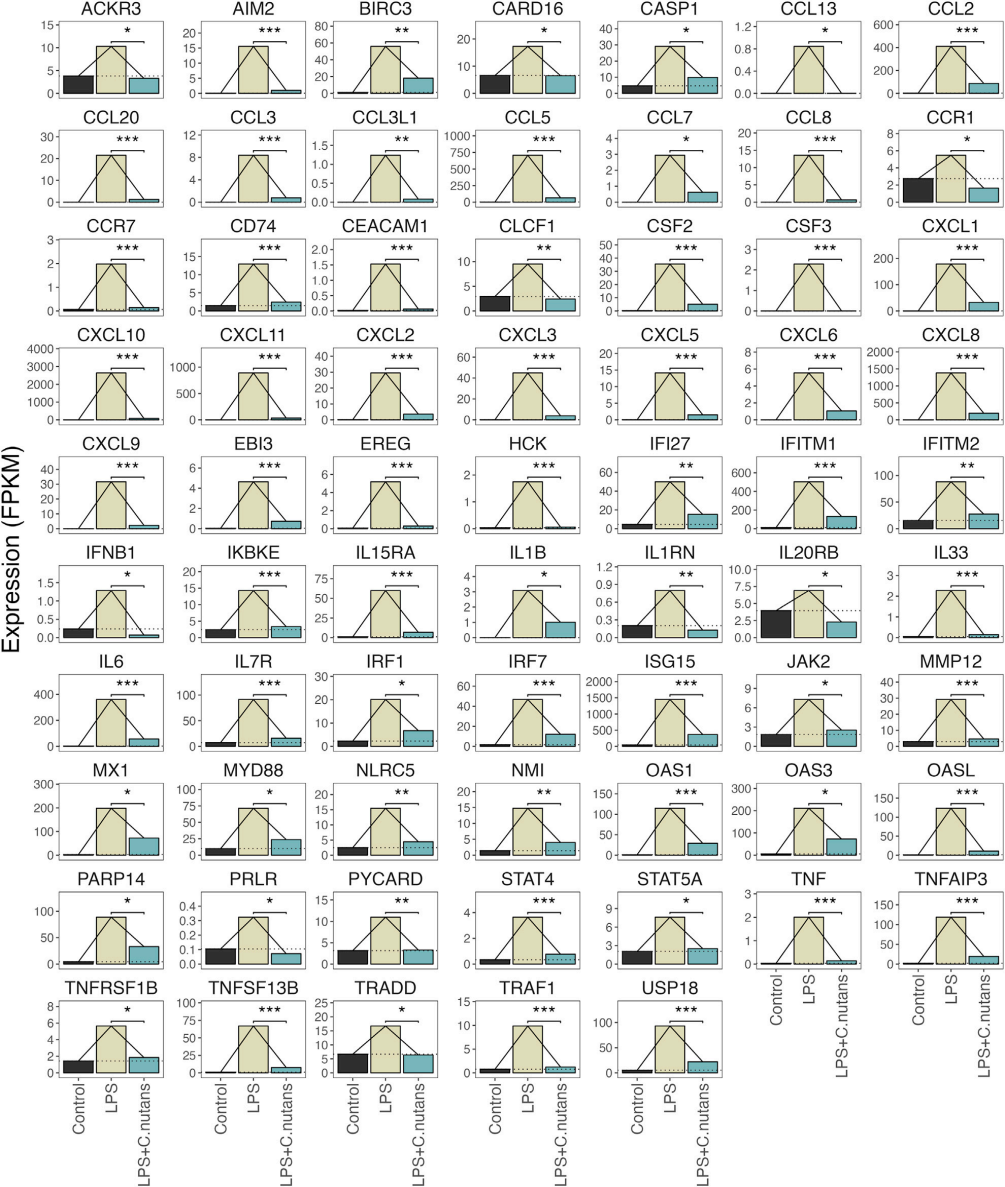
DEGs within the Cytokine-mediated signaling pathway. Expression changes in genes differentially expressed between *C. nutans* treated and LPS-treated cells in the GO term GO:0019221. Bejamini-Hochberg adjusted *p*-values for expression change between the LPS + *C. nutans* and LPS conditions are indicated: *p < 0.05, **p < 0.01, and ***p < 0.001.

To elucidate the biological processes and pathways overrepresented among these DEGs, we conducted GO-BP and KEGG analysis, focusing on the DEGs that exhibited downregulation upon *C. nutans* treatment. The top fifteen enriched terms identified through GO-BP analysis primarily encompassed processes related to immune function and inflammation (Figure 5A). These processes included cytokine-mediated signaling, regulation of response to biotic stimulus, and response to LPS. These findings were validated by KEGG analysis (Figure 5B), which revealed the top enriched pathways including cytokine-cytokine receptor interaction, the TNF signaling pathway, and the NF-κB pathway. Collectively, these data suggest that *C. nutans* treatment results in the downregulation of inflammation and immune signaling pathways stimulated by LPS. To explore these changes in more detail, we individually examined the gene expression of the downregulated DEGs within GO term “cytokine-mediated signaling” (Figure 6). Within this gene set, we observed the upregulation of numerous cytokines, chemokines, and interleukins, as well as proteins of the NF-κB, TNF and STAT families following LPS treatment. Some of those upregulations could be significantly suppressed by *C. nutans* treatment. Notably, the expression of *CXCL10*, which exhibited the highest expression levels following LPS stimulation, was validated by the qPCR data (Supplementary Figure S1). The result showed that treatment with 100 µg/mL of *C. nutans* led to a significant reduction in *CXCL10* expression, down to 0.03-fold. Taken together, *C. nutans* treatment was observed to downregulate inflammatory and immune signaling pathways activated by LPS treatment.

## 4 Discussion

Diabetes with high glucose levels pose an increased risk factor for gingiva inflammation, contributing to the progression of periodontitis. Effective treatment to decrease inflammation could potentially prevent periodontitis and associated complications in diabetes patients. Accordingly, we investigated the potential of *C. nutans* extract to attenuate inflammation induced by LPS stimulation under high-glucose conditions.

Our prior research has demonstrated that *C. nutans* extract exhibited significant antibacterial, anti-apoptosis, and anti-inflammatory properties in bovine endothelial cells following LPS induction (Panya et al., 2020; Thongyim et al., 2023). Based on these findings, we hypothesized that *C. nutans* extract could effectively suppress inflammation induced by various bacterial species, as LPS commonly activates signaling pathways that promote inflammation in the host cells. LPS, an outer-membrane component of Gram-negative bacteria, robustly triggers acute inflammation response by releasing the pro-inflammatory cytokine and activating innate immune cells such as macrophages, and neutrophils. To illustrate the acute response, we assessed the gene expression of *IL6* and *IL8* after the stimulation of LPS derived from *P. gingivalis*, particularly in high-glucose conditions (Figure 1) highlighting the influence of high-glucose levels on the inflammation response. Our result aligned with a study by Chiu et al., in 2017, which reported a significant increase of *IL6* and *IL8* due to LPS from *P. gingivalis* under high-glucose conditions compared with normal glucose (5 mM) (Chiu et al., 2017). Furthermore, LPS stimulation induced the expression of *p65*, *IκBα*, and *TLR4* mRNA, underscoring the involvement of the NF-κB pathway in the inflammation response (Chiu et al., 2017).

Previously, potential bioactive compounds from the same *C. nutans* extract with this work has been identified to be glyceryl 1,3-distearate (C_39_H_76_O_5_), kaempferol 3-O-feruloyl-sophoroside 7-O-glucoside (C_43_H_48_O_24_), and hydroxypthioceranic acid (C_46_H_92_O_3_) (Panya et al., 2020; Thongyim et al., 2023). Here, we have demonstrated the anti-inflammatory effect of *C. nutans* extract in reducing the gene expression of *IL6* and *IL8*, along with a significant decrease in corresponding secreted proteins upon LPS stimulation (Figures 2B,D). In contrast, the bioactive compound glyceryl 1,3 distearate exhibited no impact on the expression of *IL6* and *IL8* (Figure 2C). Glyceryl 1,3 distearate, along with other compounds, was identified as a major bioactive compound of *C. nutans* extract by our group using LC-MS/MS (Panya et al., 2020). In a recent study, our group validated the inhibitory effect of glyceryl 1,3 distearate in suppressing the *IL1β*, *IL6*, *IL8,* and *CXCL3* overexpression induced by LPS from *Escherichia coli,* a bacterium causing bovine mastitis (Thongyim et al., 2023). However, in the current study, glyceryl 1,3 distearate failed to inhibit the overexpression of *IL6* and *IL8* in the periodontitis model. It is plausible that the inflammation response in bovine mastitis and periodontitis differs, particularly in terms of the upstream signaling cascades. To provide a more comprehensive understanding, we could also investigate the changes in anti-inflammatory cytokines, such as IL-10, which has a role in suppressing the expression of pro-inflammatory cytokines (Steen et al., 2020). This information would offer an overview of the upstream signaling pathways involved.

Zhang and colleagues reported in 2008 that LPS derived from *P. gingivalis* induced IL1β, IL6, and TNF-α production in a manner distinct from that of *E. coli* in THP-1 cells (Zhang et al., 2008). Treatment of THP-1 cells with specific neutralizing antibodies to block the signaling cascade after LPS stimulation revealed the activation of IL6, IL1β and TNF-α production induced by *E. coli*-derived LPS primarily occurs via the TLR4 signaling pathway, while TLR2 signaling was crucial for the induction by *P. gingivalis*-derived LPS (Zhang et al., 2008). The bias toward TLR signaling may explain the difference in the effect of glyceryl 1,3 distearate if it specifically inhibited TLR agonist. On the other hand, the crude extract, containing a mixture of bioactive compounds, may offer a broad spectrum of inhibition, effectively against both to *E. coli* and *P. gingivalis-*derived LPS, highlighting the advantage of using crude extract over the single pure compound. Aside from glyceryl 1,3 distearate, several compounds have been reported to contribute to the pharmacological activity of *C. nutans* including anti-inflammatory, antiviral, antioxidant, and anti-diabetic activities (Alam et al., 2016; Zulkipli et al., 2017; Nisar and Khan, 2020)*.* C-glycosyl flavones, such as schaftoside, isovitexin, orientin, and vitexin, identified in the leave and stem of *C. nutans,* particularly in the *n*-butanol and water-soluble portions of the methanolic extract, have been reported to contribute the anti-inflammatory activity (Tuntiwachwuttikul et al., 2004; Ong et al., 2022). Quercetin and kaempferol were identified in *C. nutans* leaf extract by using HPLC (Nisar and Khan, 2020). Interestingly, quercetin has been reported to inhibit the production protein of IL1β, IL6, IL8, and TNF-α by reducing NF-κB signaling pathway in LPS-stimulated HGFs cells (Xiong et al., 2019). However, in our HPLC analysis, we did not detect the presence of quercetin in our *C. nutans* extract (Supplementary Figure S2) suggesting that the anti-inflammatory effects observed in our experiment are likely contributed by other substances, distinct from glyceryl 1,3 distearate and quercetin. We have then further investigated other potential compounds and HPLC revealed the existence of gallic acid in *C. nutans* extract (Supplementary Figure S2; Supplementary Tables S2,S3). This may suggest that acid is one of the key compounds that contributed to the anti-inflammatory property of *C. nutans*. Notably, the effect of gallic acid treatment of inflammatory periodontal membrane stem cells extracted from periodontitis tissues (i-PDLSCs) has been reported recently to reduce oxidative stress, increase mitochondria membrane potential and glucose aerobic metabolism levels leading to the improvement of osteodifferentiation-promoting ability of i-PDLSCs (Dai et al., 2022). It is widely recognized that oxidative stress and inflammation are interconnected and play significant roles in various diseases. Nevertheless, to thoroughly understand the anti-inflammatory impact of gallic acid in alleviating inflammation associated with periodontitis, further investigation is needed.

The binding of LPS to Toll-like receptors (TLR) stimulates the activation of multiple signaling cascades, including MAPK, JAK/STAT3, NF-κB and AP-1 mediated by intermediate adapters such as MyD88, MyD88-like adapter (Mal), TIR-domain-containing adapter-inducing interferon-β (TRIF) and TRIF-related adapter molecule (TRAM). These cascades promote the inflammation response in various host cell types (Liu et al., 2018; Palsson-McDermott and O'Neill, 2004). Notably, the activation of signaling pathways varied in a cell-type-specific manner. For instance, a study by Liu in 2018 compared the signaling response to LPS exposure between lung mucoepidermoid carcinoma cell line (H292) and leukemia monocytic cell line (THP-1) (Liu et al., 2018). This study revealed that LPS exposure activated NF-κB and AP-1 signaling in H292 cells, while in THP-1 cells, it activated NF-κB and JAK/STAT3 signaling (Liu et al., 2018). In our investigation, we confirmed the activation of NF-κB signaling in LPS-treated HGF-1 cells through NF-κB nuclear translocation and an immunoblotting assay (Figure 3; Supplementary Figure S3). We also demonstrated the ability of *C. nutans* extract to reduce NF-κB nuclear translocation and decrease the levels of phosphorylated IκBα and NF-κB p65 proteins (Figure 3; Supplementary Figure S3A). These results suggest that the anti-inflammation properties of *C. nutans* extract were through NF-κB signaling pathway. The inhibition of NF-κB signaling led to a significant reduction of COX2 protein levels, emphasizing the attenuated inflammatory response in *C. nutans-*treated cells (Figure 3C; Supplementary Figure S3C).

To gain deeper insights into the molecular mechanisms underlying the anti-inflammatory activity of *C. nutans* in periodontitis*,* we conducted RNA-seq transcriptomic analysis on HGF-1 subjected to various treatments, including DMSO as a control, LPS treatment, and LPS treatment in the presence of *C. nutans* extract. RNA-seq is a high-throughput sequencing technique that quantifies and profiles the expression levels of RNA, providing insights into gene expression, alternative splicing, and transcriptome dynamics (Kukurba and Montgomery, 2015; Stark et al., 2019). A total of 622 differentially expressed genes (DEGs) were observed in response to LPS treatment, with the majority exhibiting upregulation. In the comparison between LPS + *C. nutans* and LPS conditions, 622 DEGs were identified, with most showing downregulation. These trends were further validated by PCA, where *C. nutans* appeared to mitigate the gene expression perturbations induced by LPS. The KEGG analysis unveiled remarkable alterations in genes related to cytokine-cytokine receptor interaction, the TNF signaling pathway, and NF-κB pathway (Figure 5). These included changes in the expression of cytokines, chemokines, and proteins within the NF-κB, TNF, and STAT families (Figure 6). Additionally, we observed the upregulation of MyD88 and JAK2 upon LPS treatment, suggesting that LPS-TLR binding leads to NF-κB activation. Notably, the samples used this our study were pooled from three independent experiments in a single analysis, thus the results do not include error bars but reflect variation from these experiments. However, the reduction in IL6 and IL8 gene expression, as evidenced in both our RNA-seq data and confirmed through real-time PCR results, serves as a validation of our methodology employed to investigate changes in gene expression. Furthermore, the involvement of NF-κB signaling, the pathway identified by KEGG term, was confirmed by Western blot analysis, revealing the suppression of NF-κB signaling which might contributed for IL6, IL8, and CXCL10 downregulation. Taken together, the concordant results from real-time PCR and Western blot confirmed the transcriptomics result and support its reliability. Beyond the NF-κB signaling pathway, we also observed changes in genes involved in the cytokine network associated with host-immune response in periodontitis. These genes included numbers of IL1 family (IL1 IL33, MyD88, TRAF), IL6 family (JAK, STAT), and TNF family (TNF, TRADD, TRAF, TNFRSF) (Figure 6). This cytokine network plays a crucial role in periodontitis pathogenesis, affecting the recruitment of immune cells and regulating osteoclastic activity (Pan et al., 2019). The suppression of these cytokine networks by *C. nutans* extracts underscores its potential to reduce the risk as well as slowing the progression and severity of periodontitis.

Evaluation of the effectiveness of *C. nutans* in preventing or slowing periodontitis in diabetic patients is considered a gap in our study. We demonstrated the anti-inflammatory effects of *C. nutans* on LPS-induced inflammation under high-glucose conditions, but not at the clinical trial level. A clinical trial in humans is necessary to provide evidence-based benefits of the treatment on disease outcomes. Moreover, in this work, transcriptomic analysis was employed to understand the underlying mechanisms through sequential signaling events. Nevertheless, further investigations, such as proteomics or metabolomics, are needed to support the reliability of our findings and provide more detailed information. In conclusion, our findings highlight the unique therapeutic potential of *C. nutans* extract in controlling inflammation induced by *P. gingivalis*-derived LPS under a high-glucose condition. The overall results suggest that *C. nutans* extract could be effectively utilized as both preventive and curative agent, depending on the time of application. Considering that diabetic patients have a high rate of developing periodontitis, approximately three times higher than non-diabetic patients, the application of *C. nutans* extract can prevent the onset of the disease. Moreover, our results show that *C. nutans* extract reduces inflammation, indicating its potential curative effects. Therefore, the application of *C. nutans* extract could help not only in preventing the disease but also in reducing the costs associated with curing the disease after its onset. This holds significant clinical implications for the treatment of periodontal disease in diabetic patients. However, to evaluate the potential outcome of *C. nutans* in diabetes patient, the clinical trials is needed. Moreover, several aspects could be further investigated prior to its actual use in diabetic patients, including synergistic effects of the extracts with medications used in diabetic care and its long-term effects to the patients.

## Data Availability

The original contributions presented in the study are included in the article/Supplementary Material, further inquiries can be directed to the corresponding author.
