# Supplementary figures and images for: Clinacanthus nutans extract lowers periodontal inflammation under high-glucose conditions via inhibiting NF-κB signaling pathway
Source: Front Pharmacol. 2024 Aug 13;15:1410419. doi: 10.3389/fphar.2024.1410419 (PMC11347419; doi:10.3389/fphar.2024.1410419)

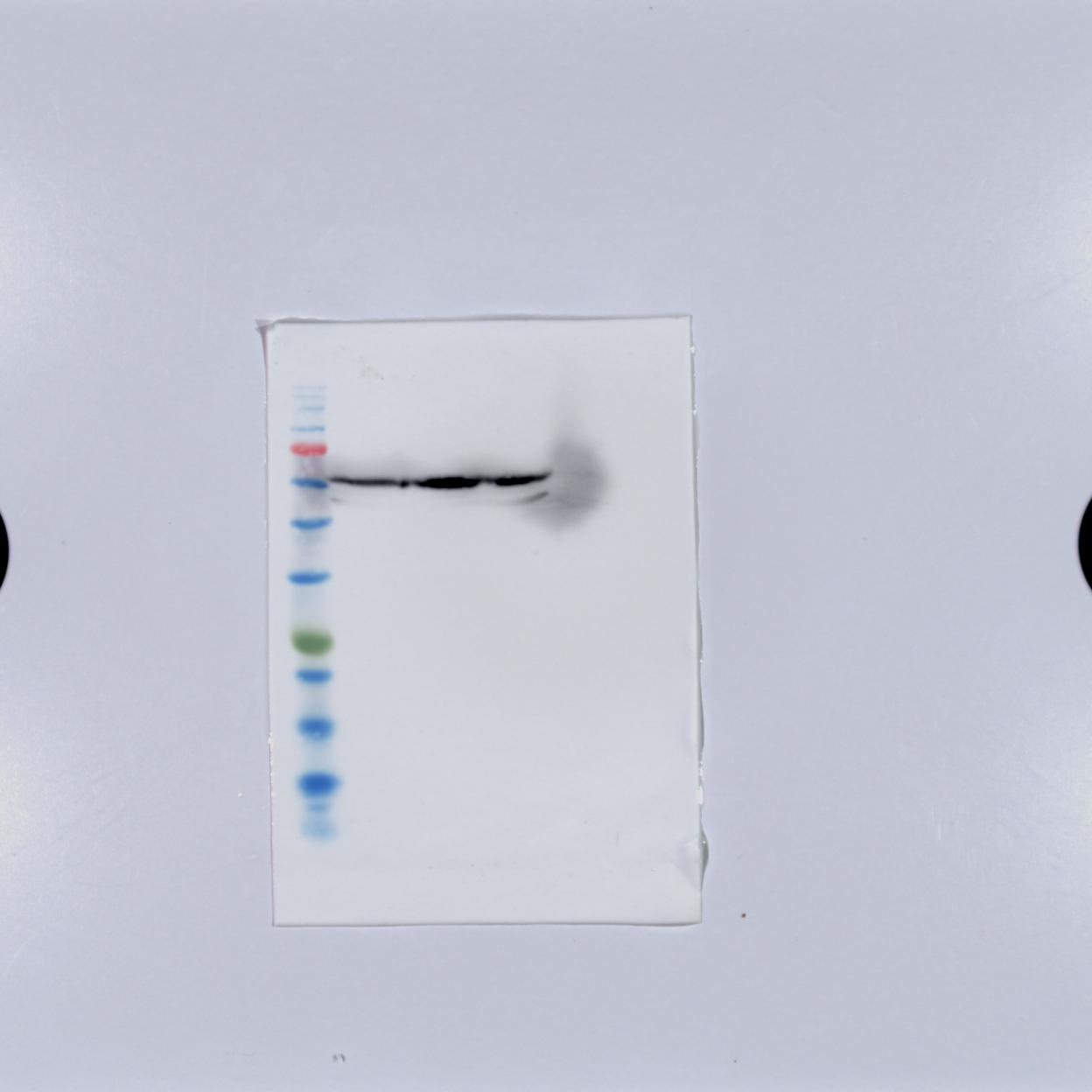

Supplement: Supplementary file 1 [file DataSheet3.zip › Supplementary Figure S3 Immunoblotting assay(B) tiff/pNF-KB.tiff]

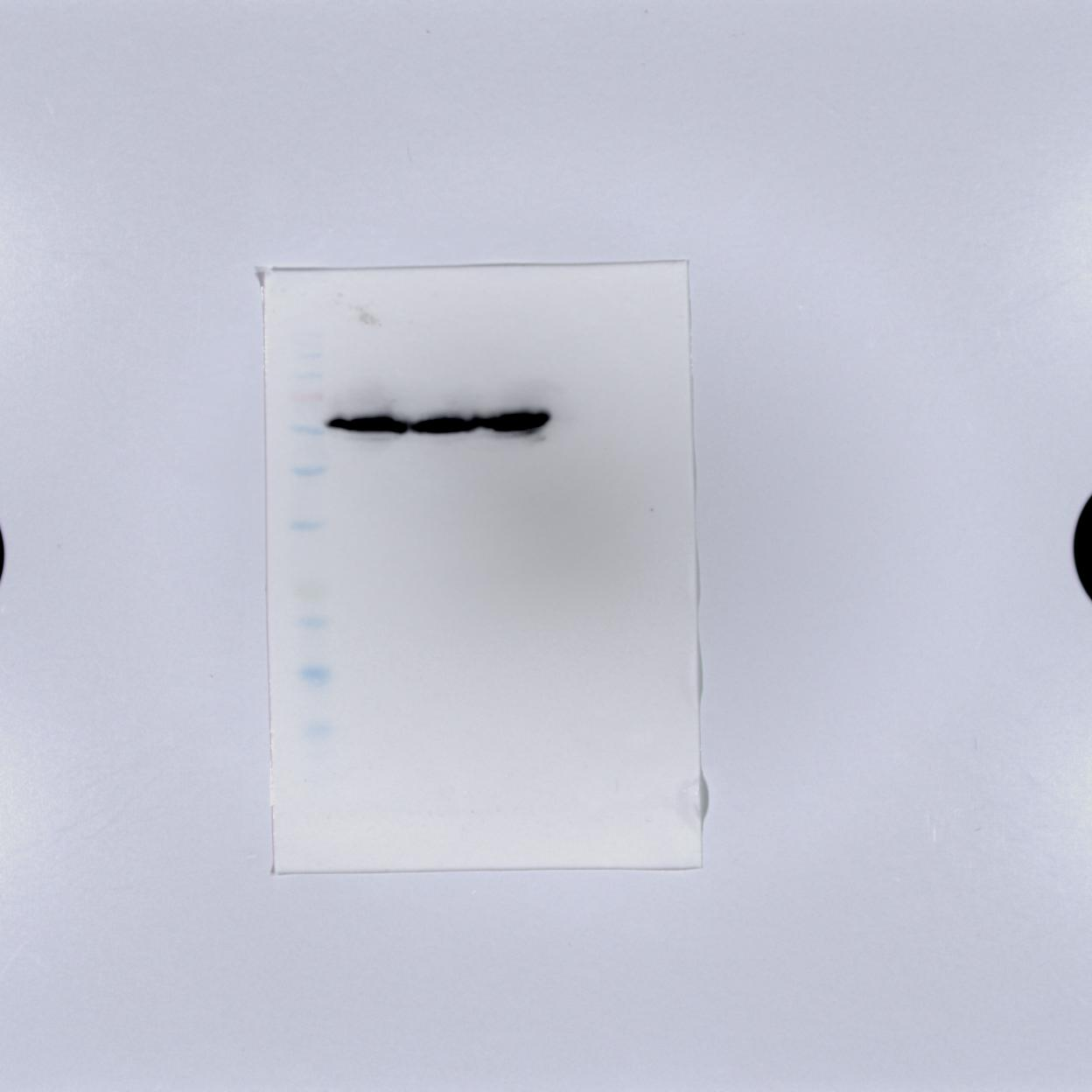

Supplement: Supplementary file 1 [file DataSheet3.zip › Supplementary Figure S3 Immunoblotting assay(B) tiff/NF-KB.tiff]

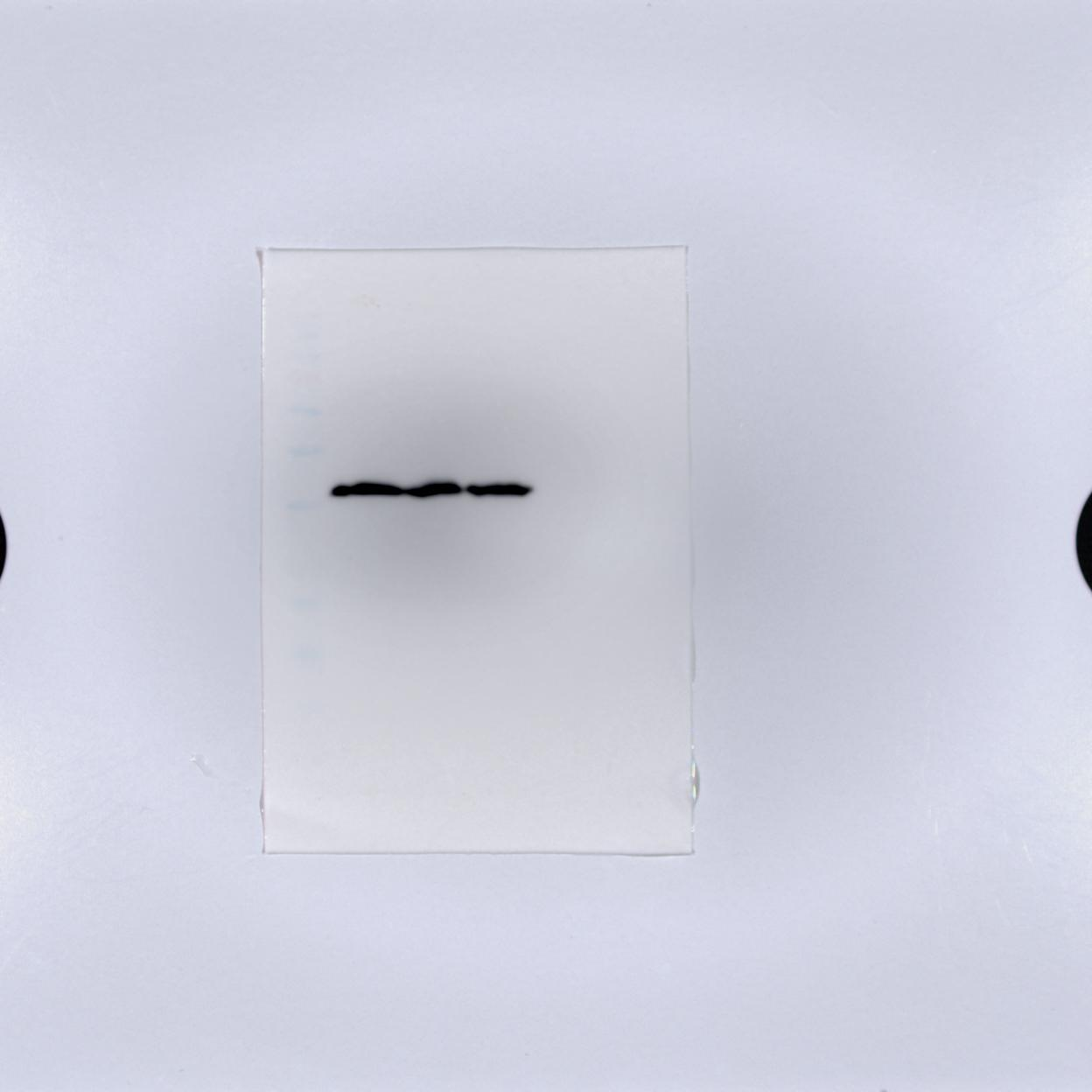

Supplement: Supplementary file 1 [file DataSheet3.zip › Supplementary Figure S3 Immunoblotting assay(B) tiff/GAP-pNF-KB,NF-KB.tiff]

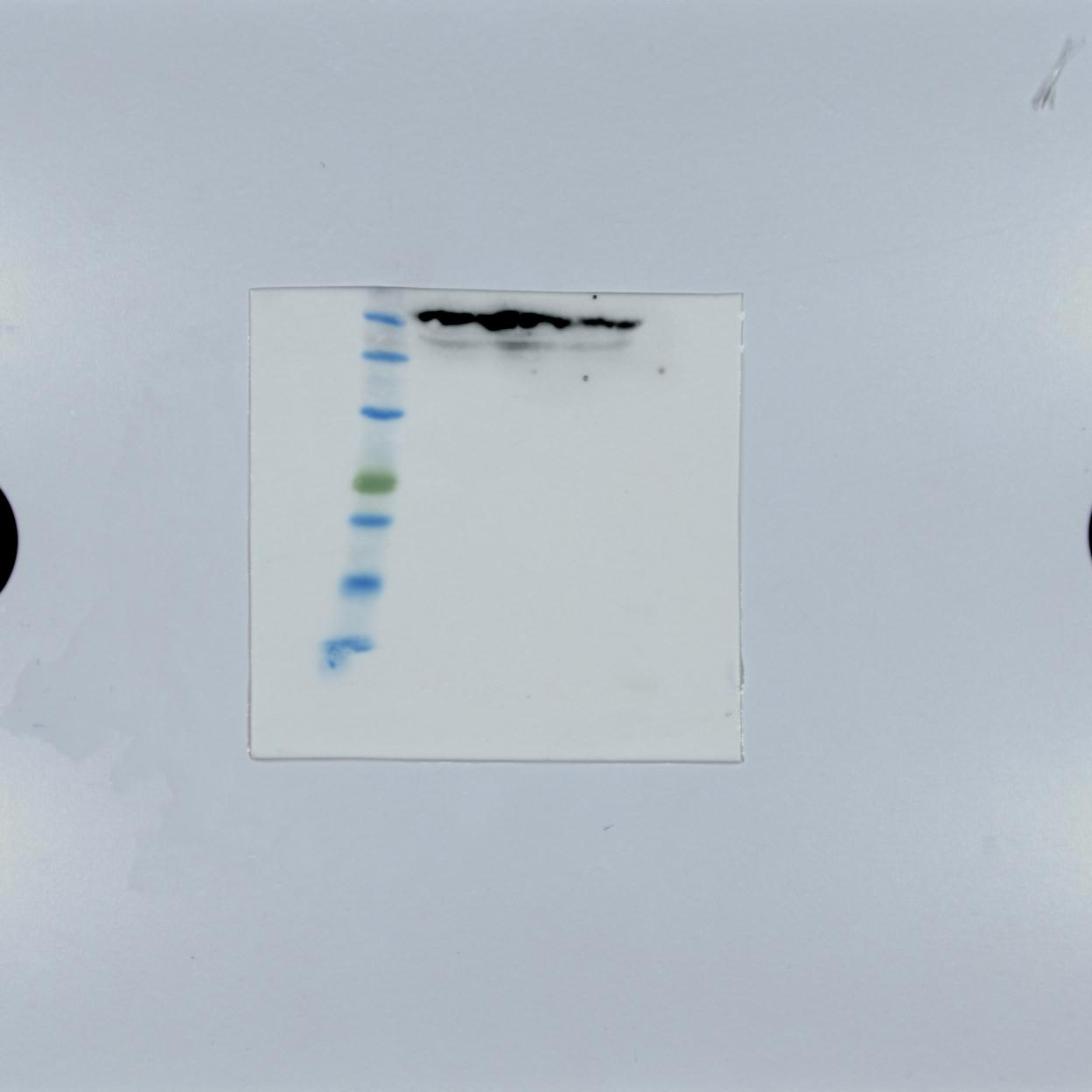

Supplement: Supplementary file 1 [file DataSheet3.zip › Supplementary Figure S3 Immunoblotting assay(B) tiff/pNF-KB2.tiff]

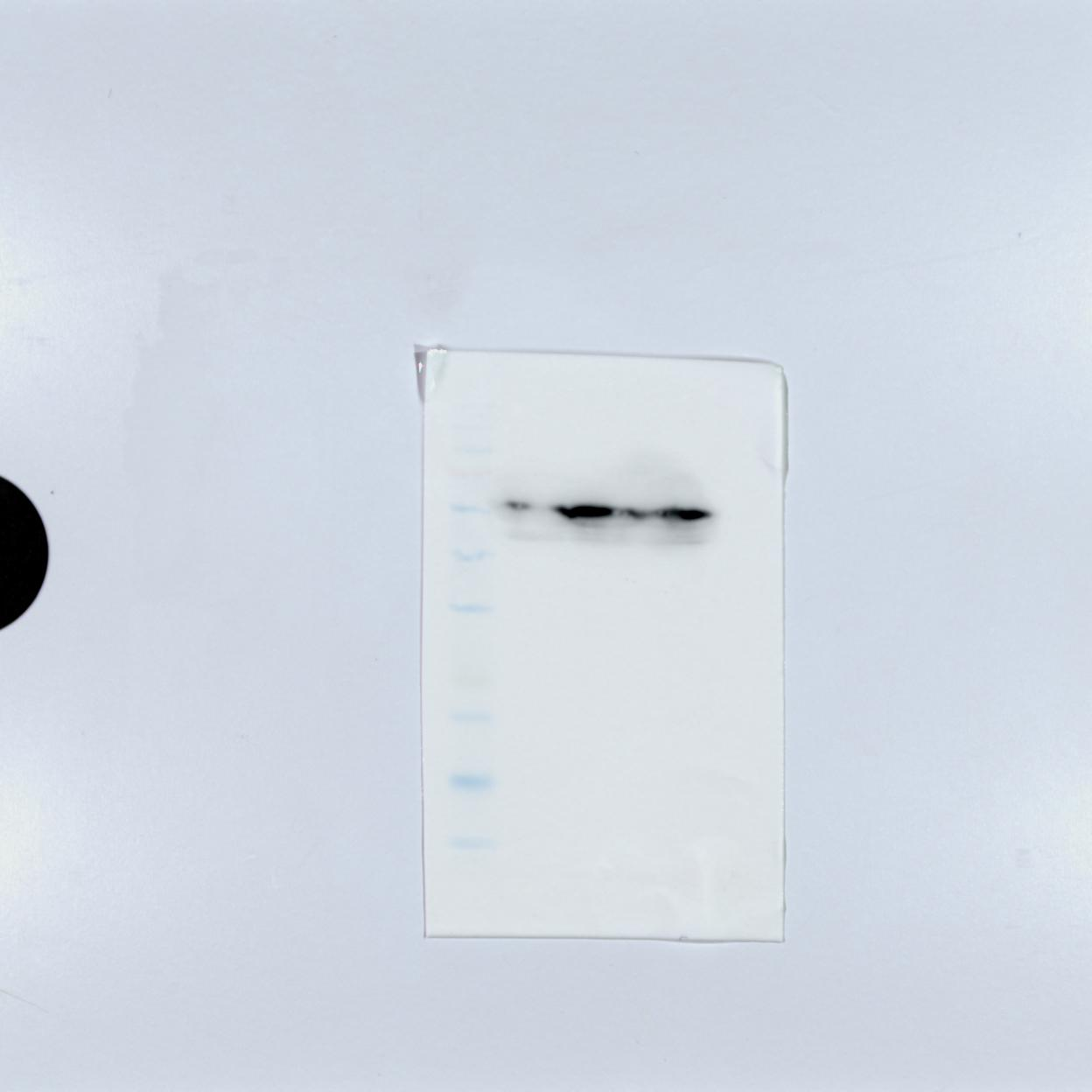

Supplement: Supplementary file 1 [file DataSheet3.zip › Supplementary Figure S3 Immunoblotting assay(B) tiff/NF-KB2.tiff]

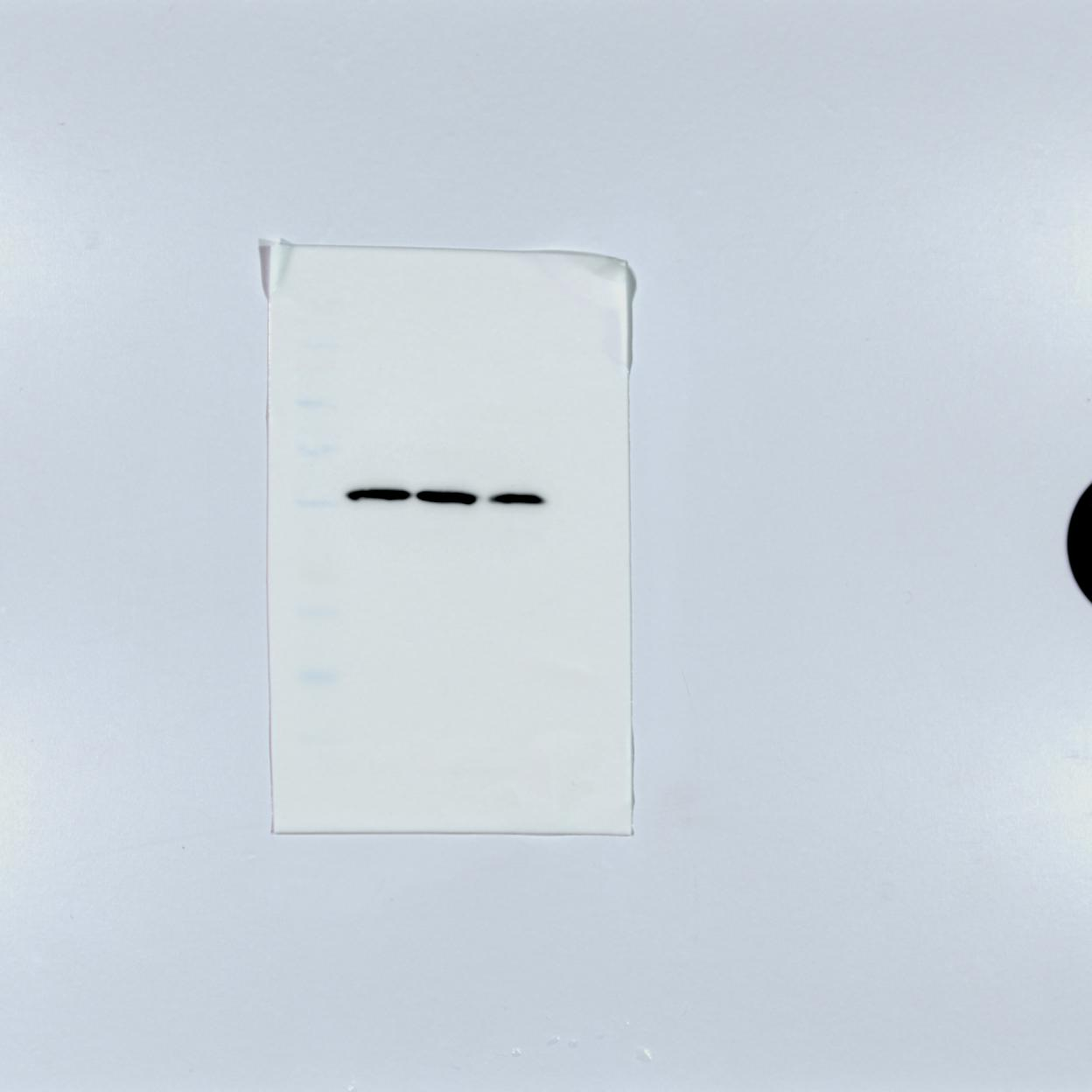

Supplement: Supplementary file 1 [file DataSheet3.zip › Supplementary Figure S3 Immunoblotting assay(B) tiff/GAP-NF-KB.tiff]

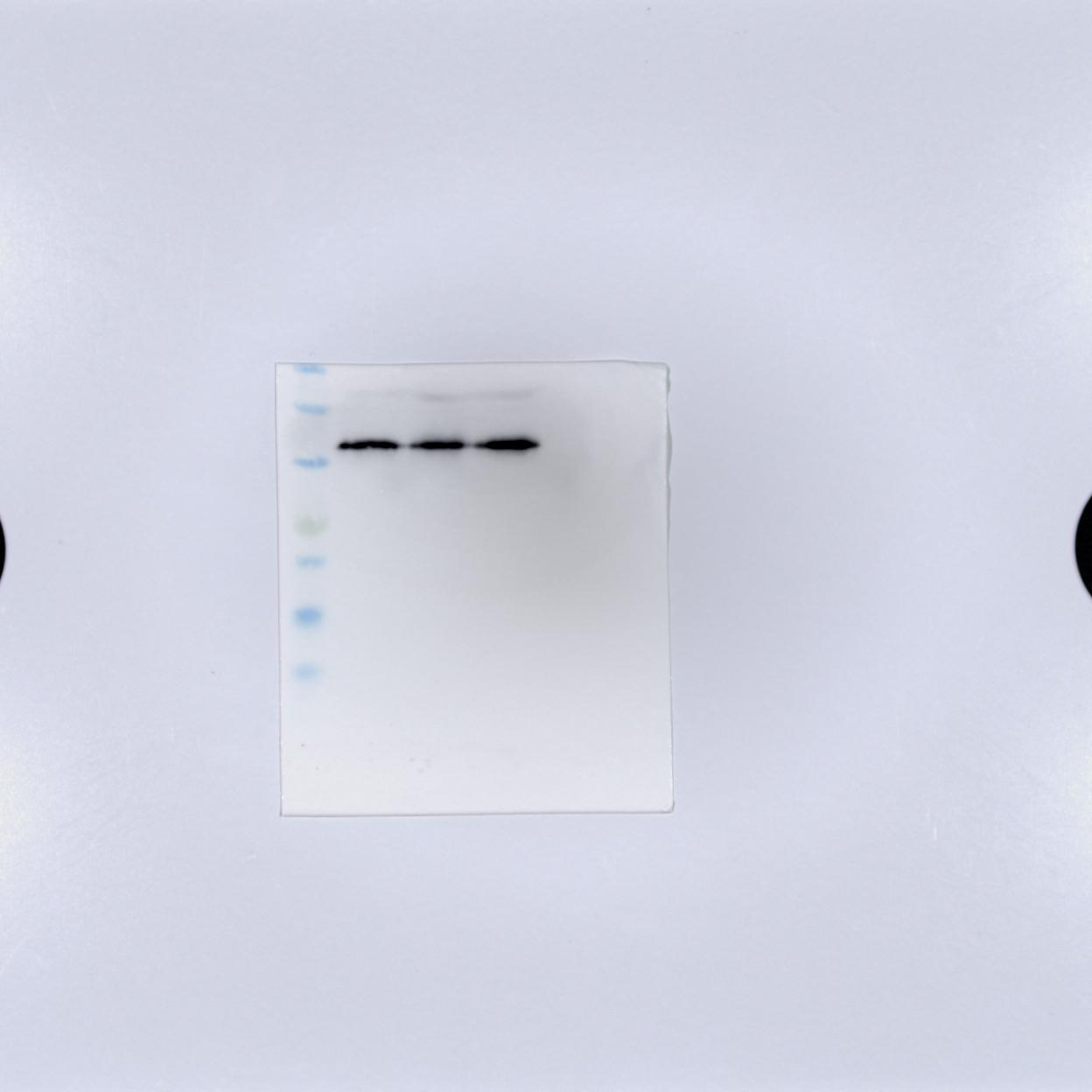

Supplement: Supplementary file 2 [file DataSheet4.zip › Supplementary Figure S3 Immunoblotting assay(A) tiff/IKB-alpha.tiff]

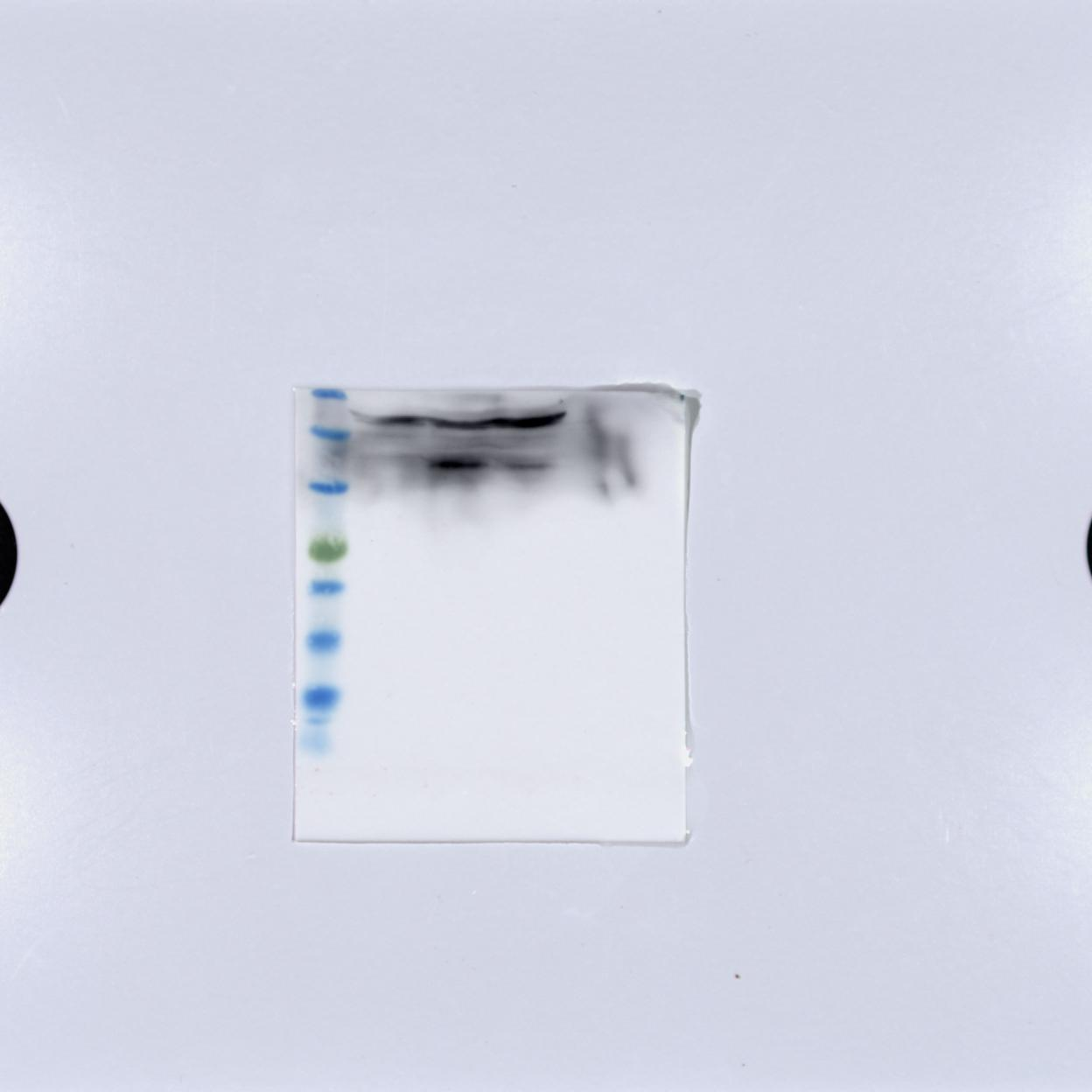

Supplement: Supplementary file 2 [file DataSheet4.zip › Supplementary Figure S3 Immunoblotting assay(A) tiff/P-IKB-alpha.tiff]

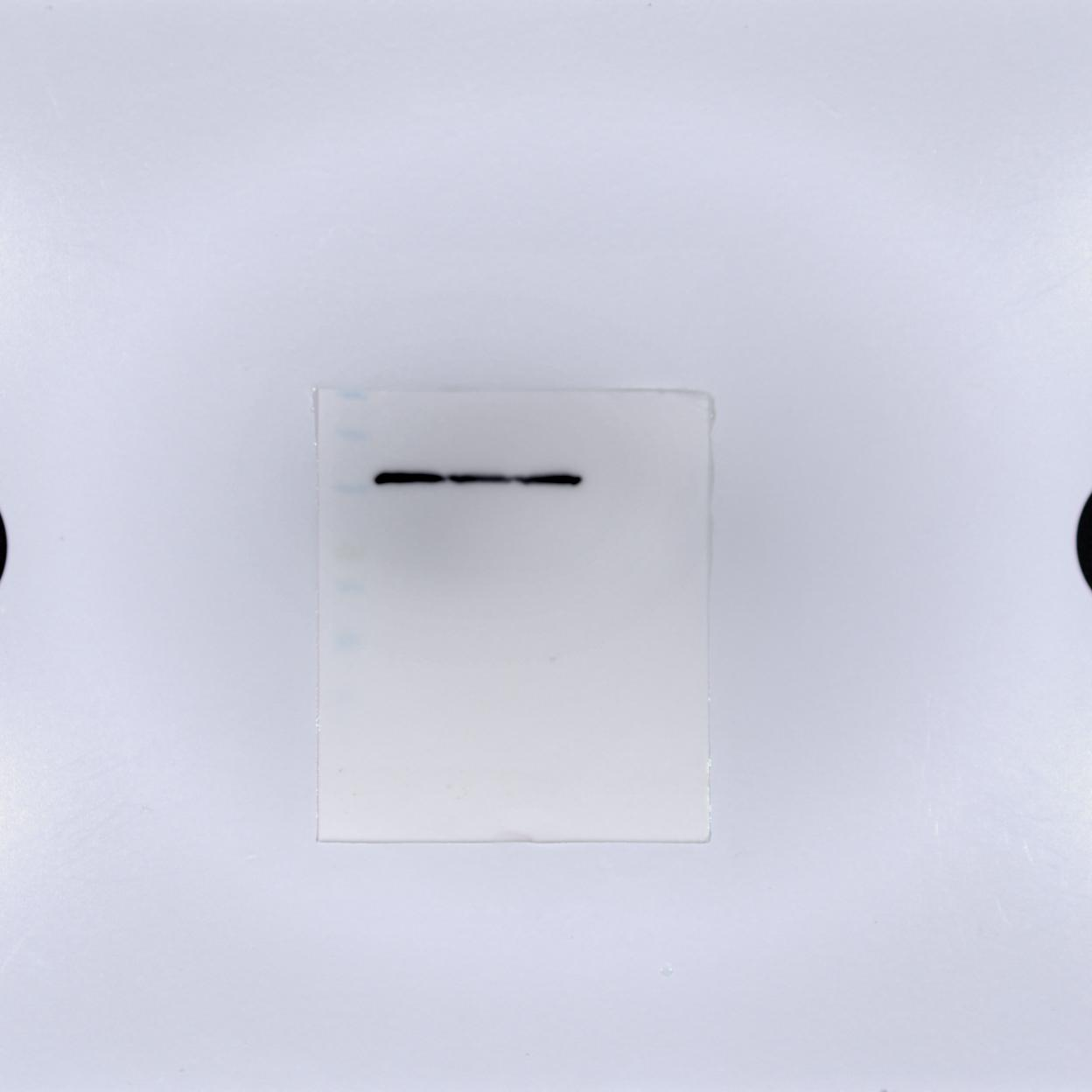

Supplement: Supplementary file 2 [file DataSheet4.zip › Supplementary Figure S3 Immunoblotting assay(A) tiff/GAP-pIKB_IKB.tiff]

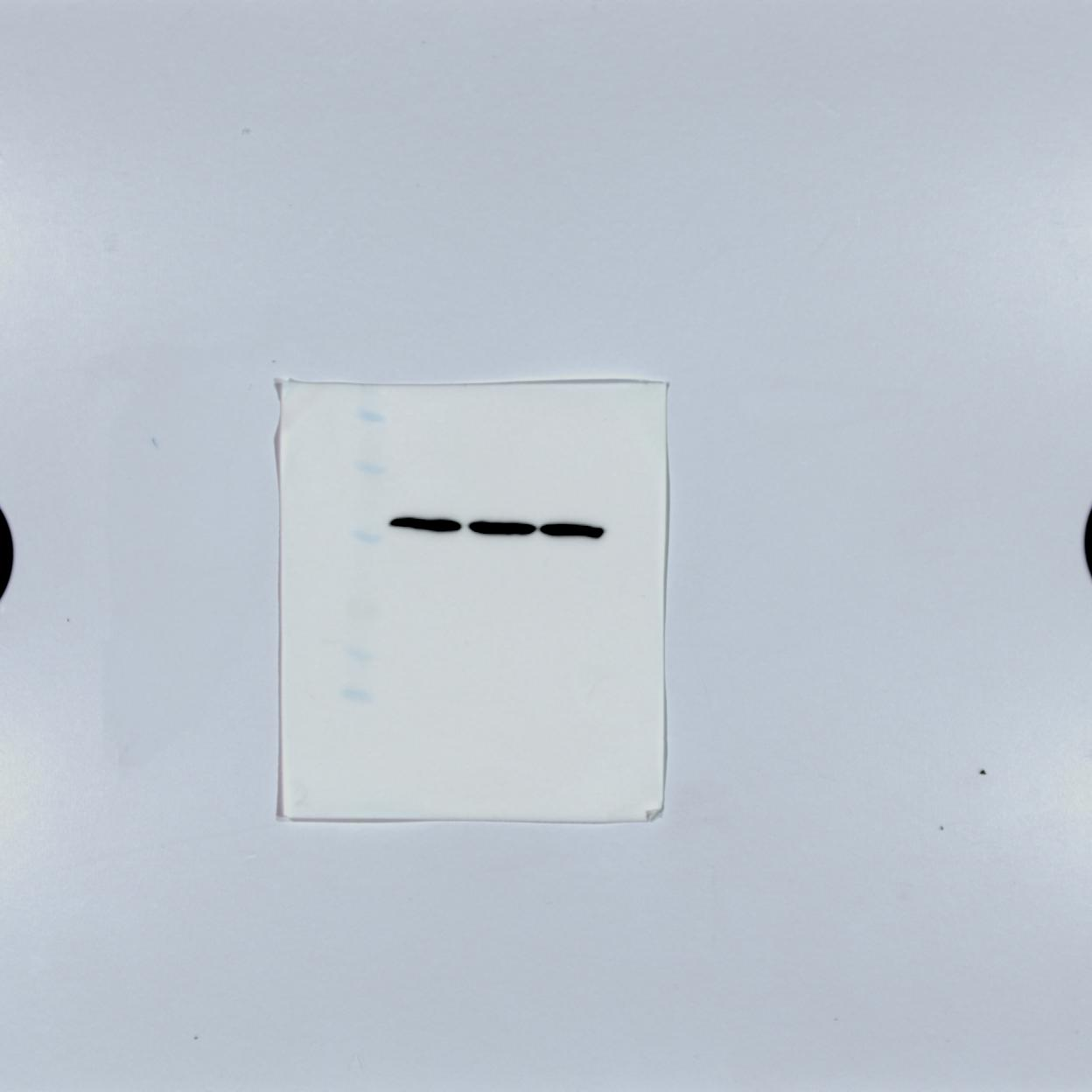

Supplement: Supplementary file 2 [file DataSheet4.zip › Supplementary Figure S3 Immunoblotting assay(A) tiff/GAP-IKB-alpha2_3.tiff]

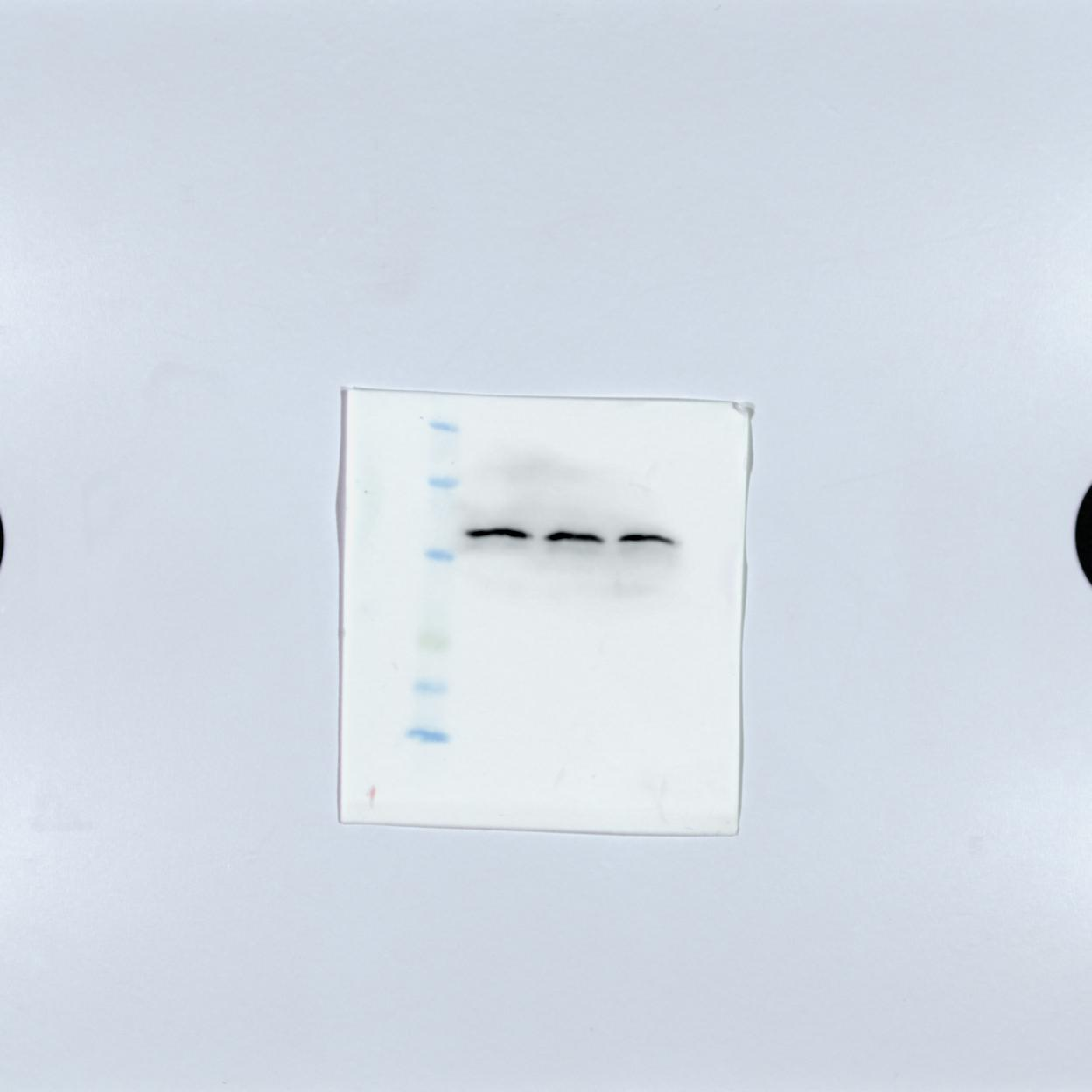

Supplement: Supplementary file 2 [file DataSheet4.zip › Supplementary Figure S3 Immunoblotting assay(A) tiff/IKB-alpha3.tiff]

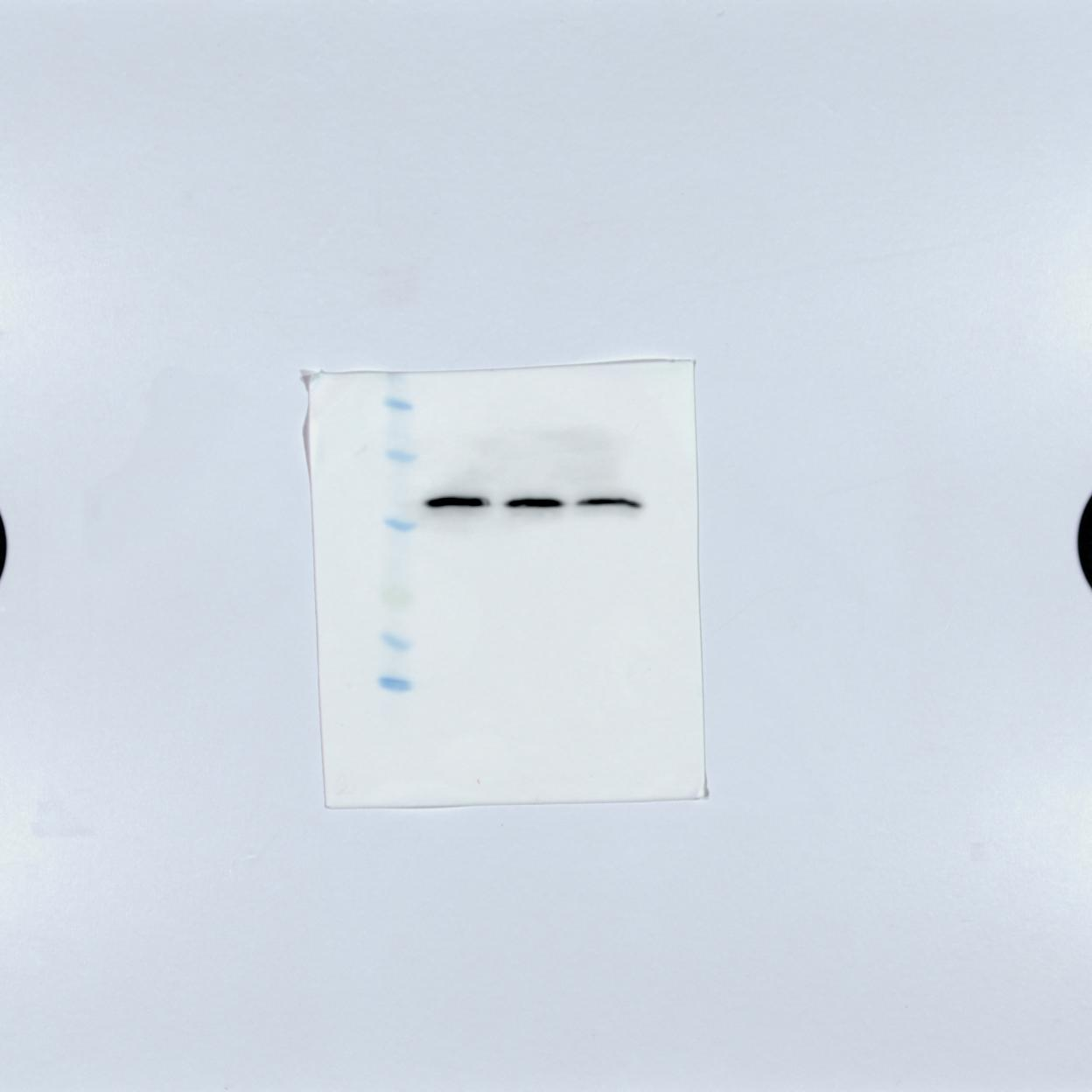

Supplement: Supplementary file 2 [file DataSheet4.zip › Supplementary Figure S3 Immunoblotting assay(A) tiff/IKB-alpha2.tiff]
