# Supplementary material for: Clinacanthus nutans extract lowers periodontal inflammation under high-glucose conditions via inhibiting NF-κB signaling pathway
Source: Front Pharmacol. 2024 Aug 13;15:1410419. doi: 10.3389/fphar.2024.1410419 (PMC11347419; doi:10.3389/fphar.2024.1410419)

Supplementary data

Clinacanthus nutans extract lowers periodontal inflammation under high-glucose conditions via inhibiting NF-κB signaling pathway

Supplementary Table S1 List of real-time PCR primers.

**Genes**	**Forward primer (5'→3')**	**Reverse primer (5'→3')**
IL6	GTACATCCTCGACGGCATC	AGGCACTGGTTCTGTGCCT
IL8	TCCTGATTTCTGCAGCTCTGT	CCAGACAGAGCTCTCTTCCA
TNF-α	TCTCCTTCCTGATCGTGGCA	GGCTACAGGCTTGTCACTCG
CXCL10	GAATCGAAGGCCATCAAGAA	AAGCAGGGTCAGAACATCCA
GADPH	CGACCACTTTGTCAAGCTCA	AGGGGTCTACATGGCAACTG

Supplementary Table S2 GC-MS/MS analysis of *C. nutans* extract. The table shows the group of chemical compounds isolated from the leaves of *C. nutans* and were identified by a comparison of their retention times (RT) with the National Institute of Standards and Technology (NIST17) libraries (Thongyim et al., 2023).

No.	Classification	Structure	Name	Molecular Formula	Retention time (RT)
1	Cyclopentanes	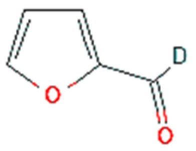	Furans	$C_5H_4O_2$	7.99
2	Disulfides	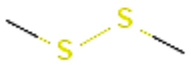	Disulfide, dimethyl	$C_2H_6S_2$	4.30
3		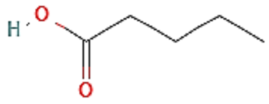	Valeric acid	$C_5H_{10}O_2$	3.73
4	Fatty Acids	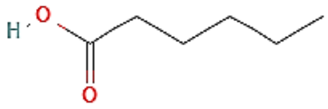	Hexanoic acid	$C_6H_{12}O_2$	4.47
5		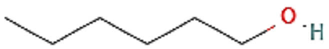	Hexanols	$C_6H_{14}O$	3.50

6		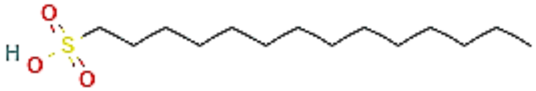	1-Tetradecanesulfonic acid	$C_{14}H_{30}O_3S$	20.67
7		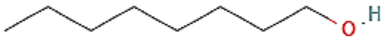	Octanol	$C_8H_{18}O$	7.56
8		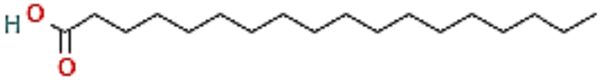	Stearic Acids	$C_{18}H_{36}O_2$	36.24
9	Fatty Acids	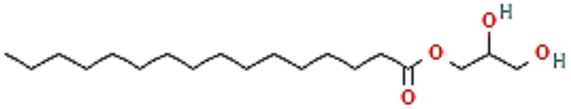	Hexadecanoic acid	$C_{19}H_{38}O_4$	45.99
10		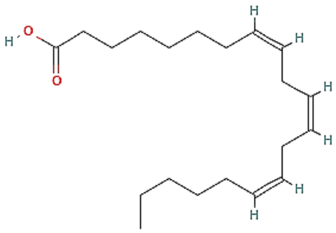	Linolenic Acids	$C_{20}H_{34}O_2$	39.64
11		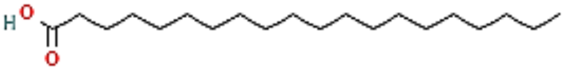	Eicosanoic Acids	$C_{20}H_{40}O_2$	40.17

12	Fatty Acids	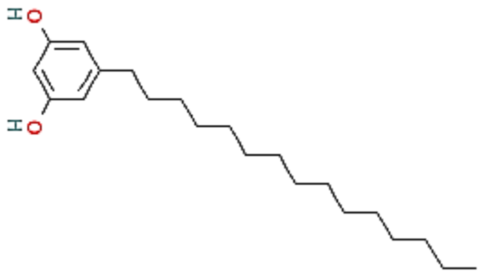	5-Pentadecylresorcinol	$C_{21}H_{36}O_2$	38.98
13		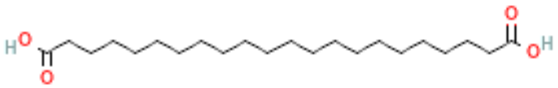	Docosanedioic acid	$C_{22}H_{42}O_4$	43.86
14		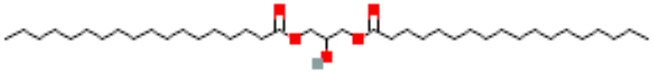	Glyceryl 1,3-distearate	$C_{39}H_{76}O_5$	49.32
15	Glycosides	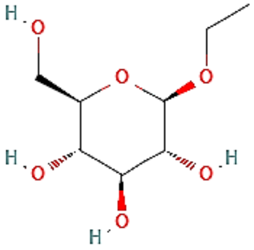	Ethyl beta-D-glucopyranoside	$C_8H_{16}O_6$	28.39

16	Terpenoids	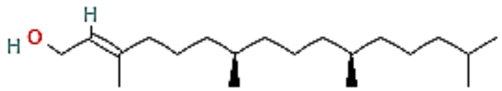	Phytol	$C_{20}H_{40}O$	38.46

*Images of the chemical structures of the bioactive compounds in *C. nutans* extract were obtained from the PubChem database

Supplementary Table S3 LC-MS/MS analysis of *C. nutans* extract. The table shows the major of chemical compounds isolated from the leaves of *C. nutans* (Panya et al., 2020).

No.	Structure	Name	Molecular Formula
1	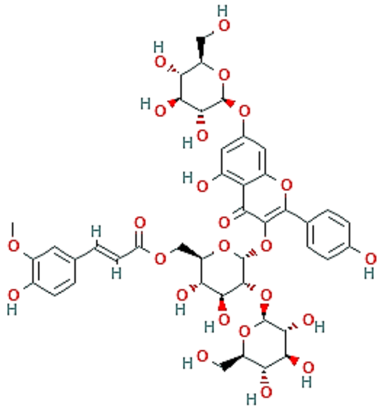	Kaempferol 3-O-feruloyl-sophoroside 7-O-glucoside	$C_{43}H_{48}O_{24}$
2	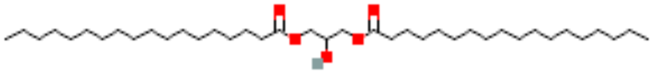	Glyceryl 1,3-distearate	$C_{39}H_{76}O_5$
3	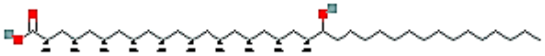	Hydroxyphthioceranic acid	$C_{46}H_{92}O_3$

*Images of the chemical structures of the bioactive compounds in *C. nutans* extract were obtained from the PubChem database

Supplementary Figure S1 Effect of *C. nutans* extracts on lowering LPS-induced *CXCL10* expression. Expression of *CXCL10* which exhibited the highest expression levels following LPS stimulation, and the effect of *C. nutans* extract on inhibiting *CXCL10* overexpression were validated by the qPCR data.

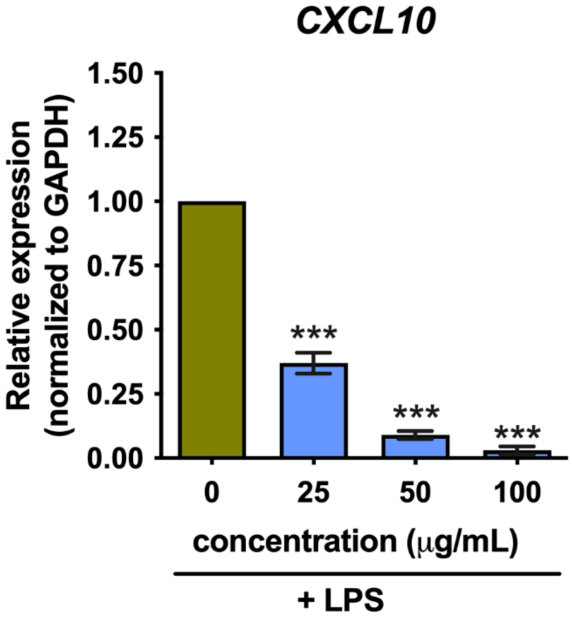

Supplementary Figure S2 HPLC analysis of *C. nutans* extract A. The gallic acid and quercetin standard compound was analyzed by using HPLC with C18 HPLC column (Reverse phase) and H_2O:methanol (70:30, V/V) mobile phase **B.** HPLC profile of *C. nutans* extract

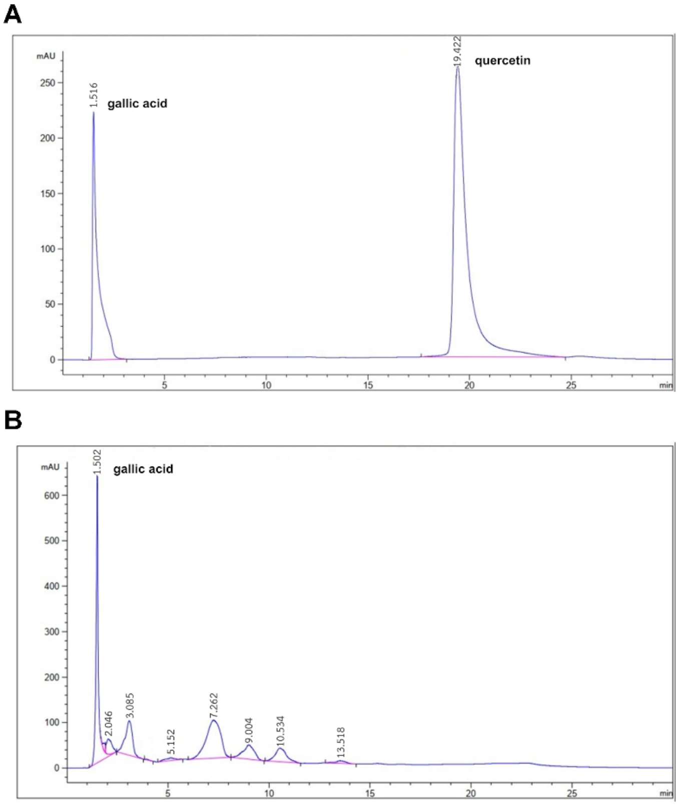

Supplementary Figure S3 LPS (10 μg/mL) was treated to HGF-1 cells in the presence or absence of *C. nutans* extracts (300 μg/mL). Immunoblotting was performed to detect the changes of IκBα and NF-κB p65, phosphorylated IκBα, phosphorylated NF-κB p65 and COX2 protein. (C = Control, LD = LPS + DMSO, and LC = LPS + *C. nutans*)

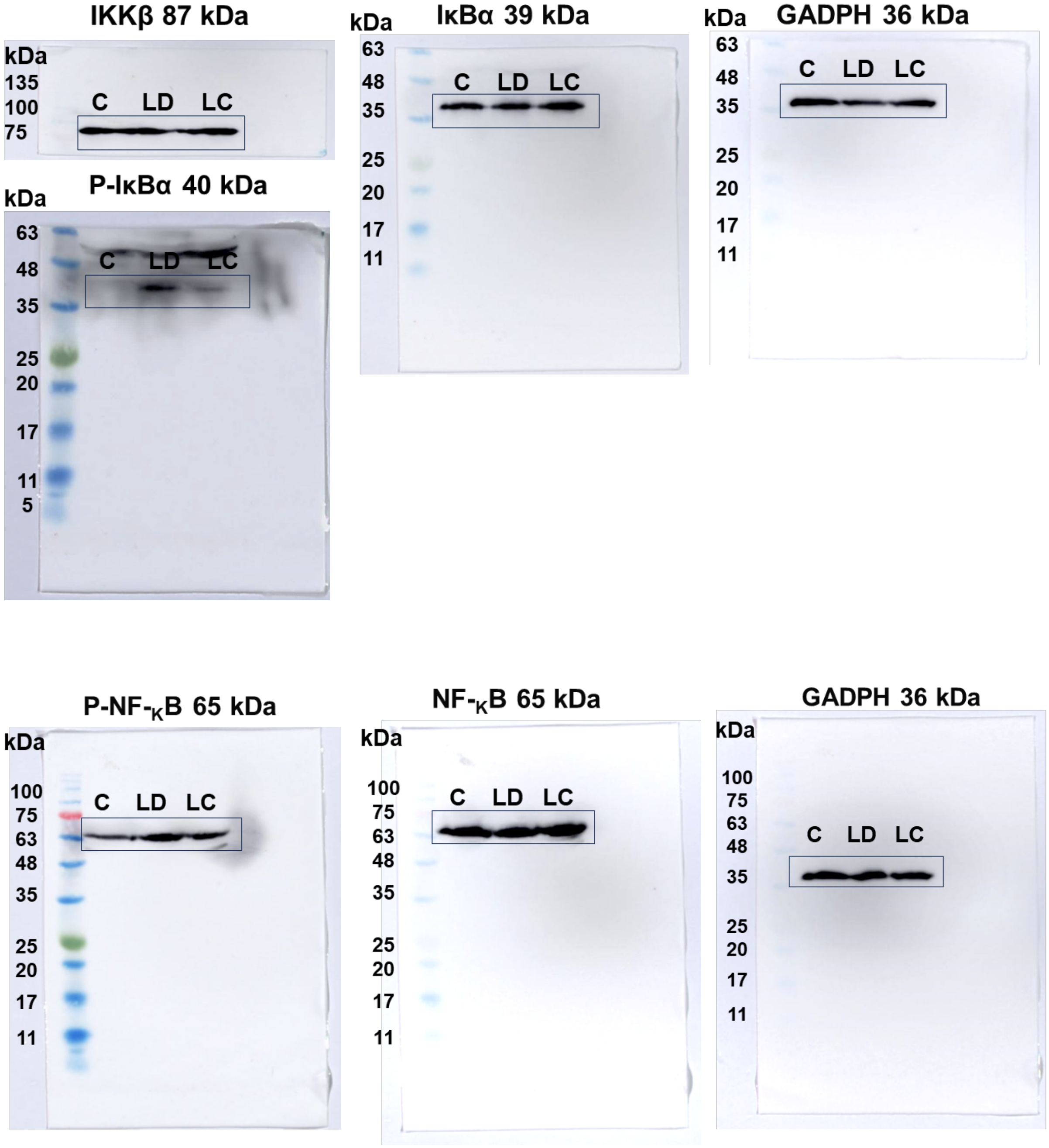

Supplementary Figure S3 LPS (10 µg/mL) was treated to HGF-1 cells in the presence or absence of *C. nutans* extracts (300 µg/mL). Immunoblotting was performed to detect the changes of IκBα and NF-κB p65, phosphorylated IκBα, phosphorylated NF-κB p65 and COX2 protein. (C = Control, LD = LPS + DMSO, and LC = LPS + *C. nutans*) (continued)

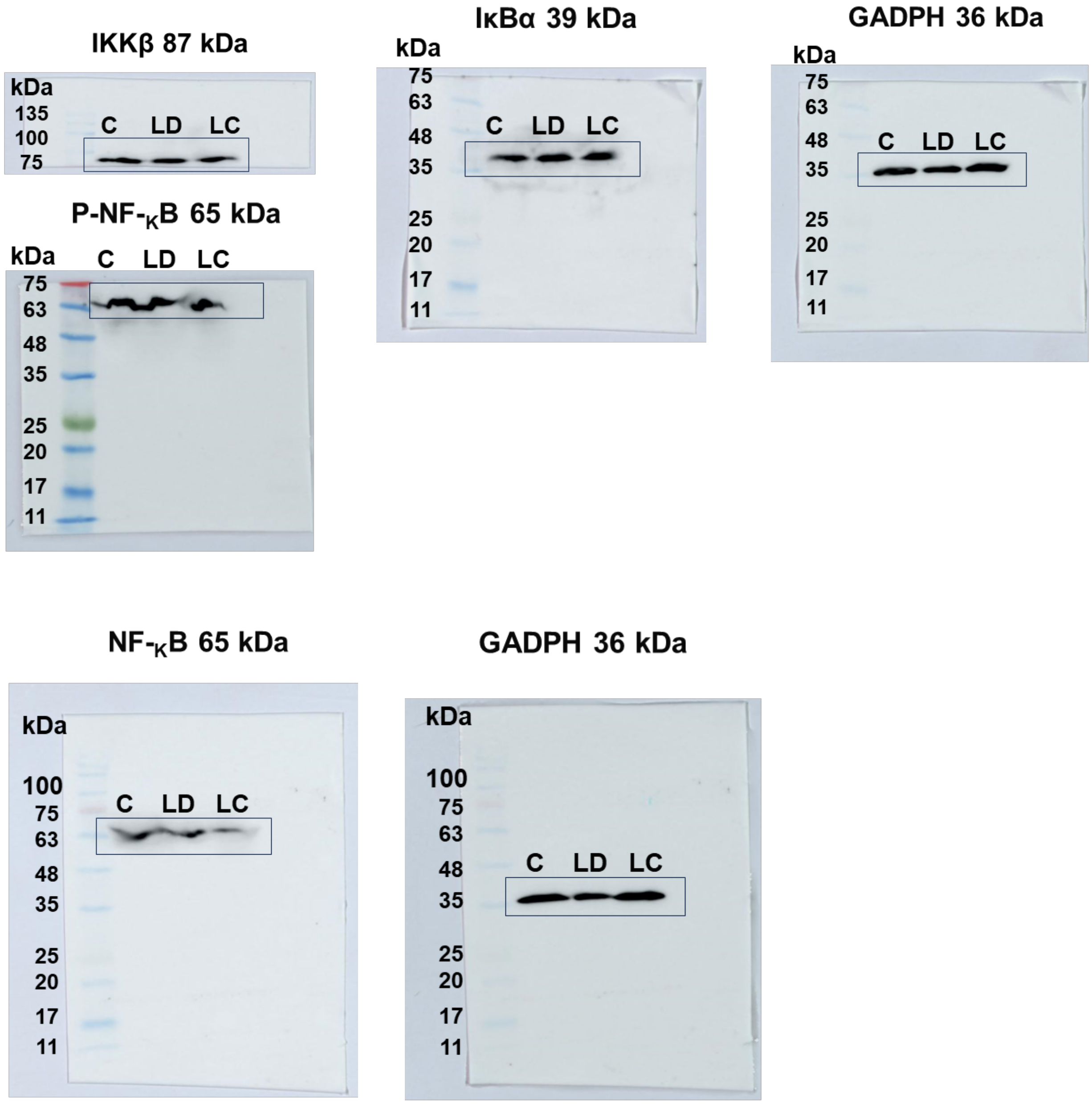

Supplementary Figure S3 LPS (10 μg/mL) was treated to HGF-1 cells in the presence or absence of *C. nutans* extracts (300 μg/mL). Immunoblotting was performed to detect the changes of IκBα and NF-κB p65, phosphorylated IκBα, phosphorylated NF-κB p65 and COX2 protein. (C = Control, LD = LPS + DMSO, and LC = LPS + *C. nutans*) (continued)

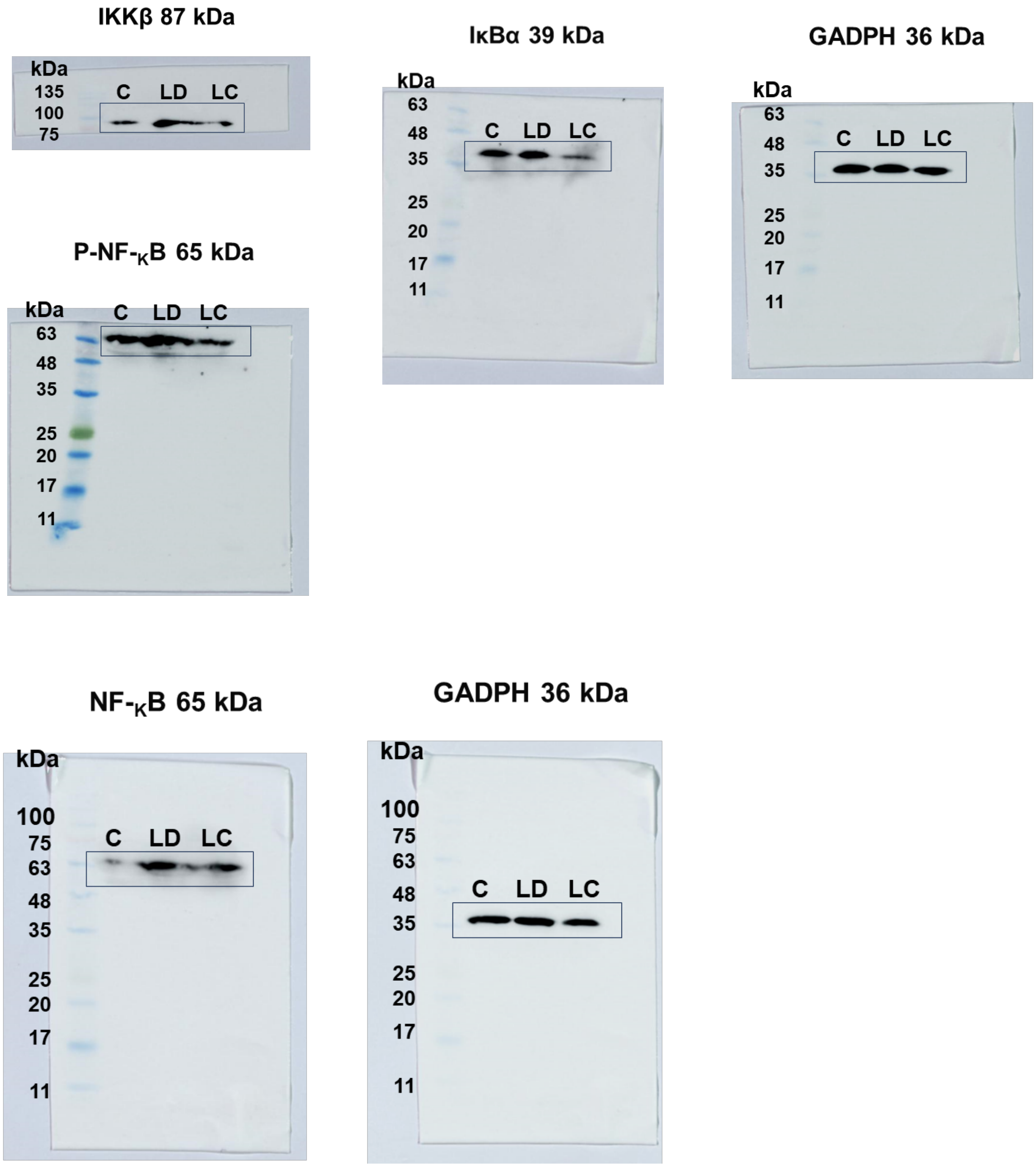

Supplementary Figure S3 LPS (10 µg/mL) was treated to HGF-1 cells in the presence or absence of *C. nutans* extracts (300 µg/mL). Immunoblotting was performed to detect the changes of IκBα and NF-κB p65, phosphorylated IκBα, phosphorylated NF-κB p65 and COX2 protein. (C = Control, LD = LPS + DMSO, and LC = LPS + *C. nutans*) (continued)

COX2 73 kDa

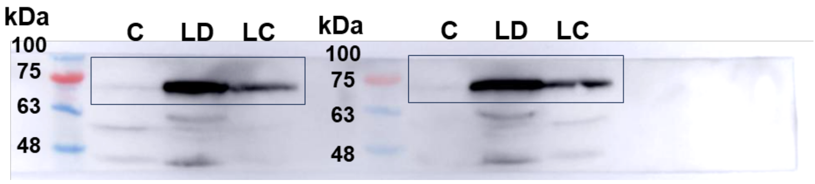

GADPH 36 kDa

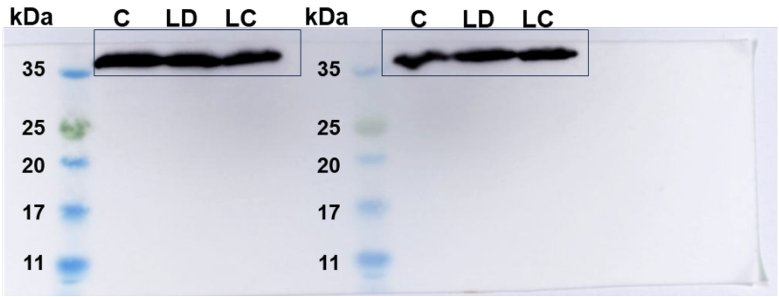

Supplementary Figure S4 LPS (10 μg/mL) was treated to HGF-1 cells in the presence or absence of *C. nutans* extracts (300 μg/mL). NF-κB nuclear translocation was monitored by IFA techniques where the nuclear area was stained by Hoechst dye (NF-κB in red and nucleus in blue with 50-μm scale bar)

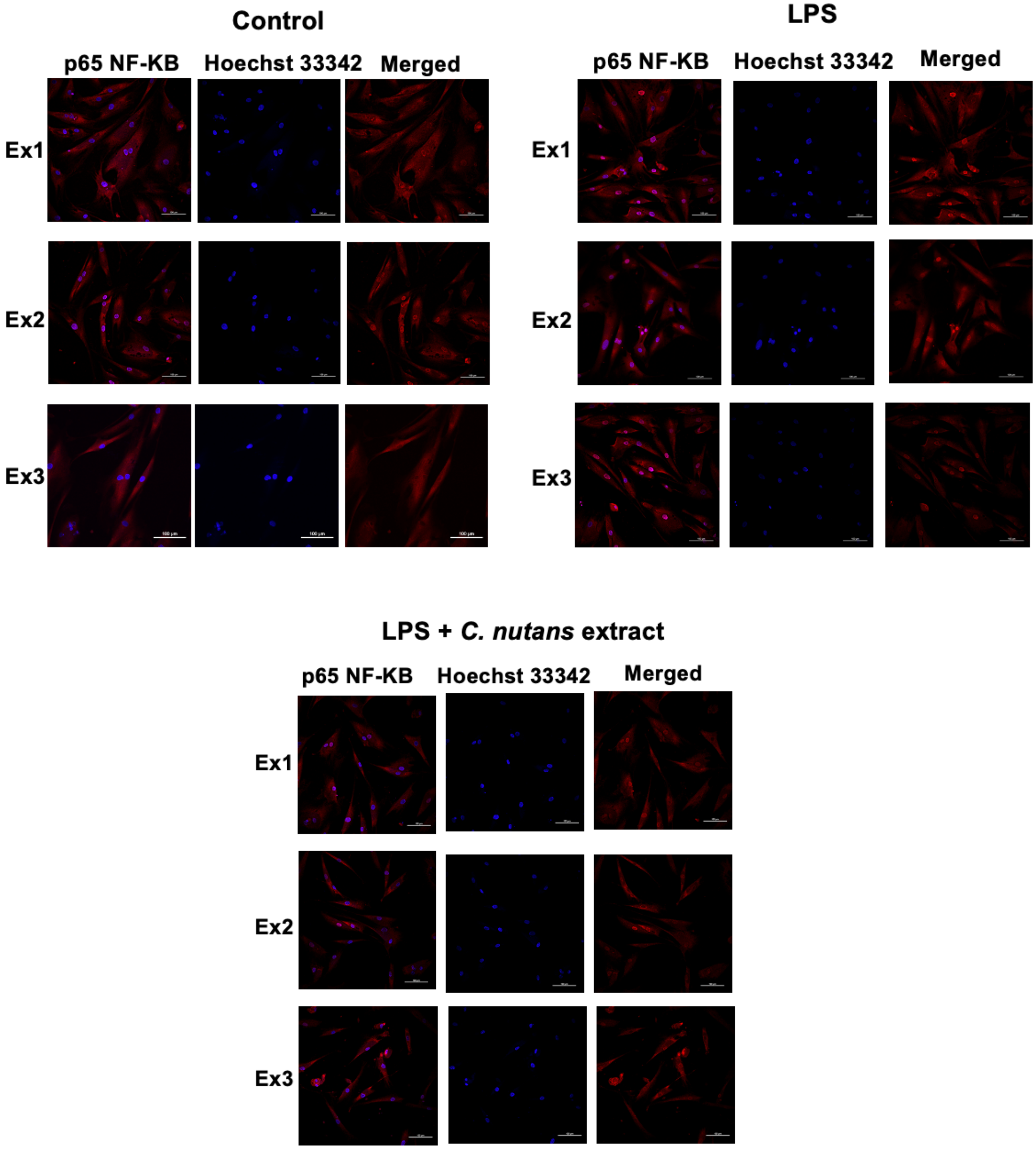

Supplement: Supplementary file 3 [file DataSheet1.pdf]
